# Spatially resolved multiomics of human cardiac niches

**DOI:** 10.1038/s41586-023-06311-1

**Published:** 2023-07-12

**Authors:** Kazumasa Kanemaru, James Cranley, Daniele Muraro, Antonio M. A. Miranda, Siew Yen Ho, Anna Wilbrey-Clark, Jan Patrick Pett, Krzysztof Polanski, Laura Richardson, Monika Litvinukova, Natsuhiko Kumasaka, Yue Qin, Zuzanna Jablonska, Claudia I. Semprich, Lukas Mach, Monika Dabrowska, Nathan Richoz, Liam Bolt, Lira Mamanova, Rakeshlal Kapuge, Sam N. Barnett, Shani Perera, Carlos Talavera-López, Ilaria Mulas, Krishnaa T. Mahbubani, Liz Tuck, Lu Wang, Margaret M. Huang, Martin Prete, Sophie Pritchard, John Dark, Kourosh Saeb-Parsy, Minal Patel, Menna R. Clatworthy, Norbert Hübner, Rasheda A. Chowdhury, Michela Noseda, Sarah A. Teichmann

**Affiliations:** 1 Wellcome Sanger Institute, Wellcome Genome Campus, Hinxton, Cambridge, UK; 2 National Heart and Lung Institute, Imperial College London, London, UK; 3 Cardiac Morphology Unit, Royal Brompton Hospital and Imperial College London, London, UK; 4 Max Delbrück Center for Molecular Medicine in the Helmholtz Association (MDC), Berlin, Germany; 5 Royal Brompton Hospital, London, UK; 6 Molecular Immunity Unit, Department of Medicine, University of Cambridge, MRC Laboratory of Molecular Biology, Cambridge, UK; 7 Würzburg Institute for Systems Immunology, Max Planck Research Group, Julius-Maximilian-Universität, Würzburg, Germany; 8 Department of Surgery, University of Cambridge, and Cambridge Biorepository for Translational Medicine, NIHR Cambridge Biomedical Centre, Cambridge, UK; 9 Translational and Clinical Research Institute, Newcastle University, Newcastle upon Tyne, UK; 10 Charité-Universitätsmedizin, Berlin, Germany; 11 German Centre for Cardiovascular Research (DZHK), Partner Site Berlin, Berlin, Germany; 12 Department of Physics, Cavendish Laboratory, University of Cambridge, Cambridge, UK

**Keywords:** Computational biology and bioinformatics, Next-generation sequencing, Transcriptomics, Cells

## Abstract

The function of a cell is defined by its intrinsic characteristics and its niche: the tissue microenvironment in which it dwells. Here we combine single-cell and spatial transcriptomics data to discover cellular niches within eight regions of the human heart. We map cells to microanatomical locations and integrate knowledge-based and unsupervised structural annotations. We also profile the cells of the human cardiac conduction system^1^. The results revealed their distinctive repertoire of ion channels, G-protein-coupled receptors (GPCRs) and regulatory networks, and implicated *FOXP2* in the pacemaker phenotype. We show that the sinoatrial node is compartmentalized, with a core of pacemaker cells, fibroblasts and glial cells supporting glutamatergic signalling. Using a custom CellPhoneDB.org module, we identify trans-synaptic pacemaker cell interactions with glia. We introduce a druggable target prediction tool, drug2cell, which leverages single-cell profiles and drug–target interactions to provide mechanistic insights into the chronotropic effects of drugs, including GLP-1 analogues. In the epicardium, we show enrichment of both IgG^+^ and IgA^+^ plasma cells forming immune niches that may contribute to infection defence. Overall, we provide new clarity to cardiac electro-anatomy and immunology, and our suite of computational approaches can be applied to other tissues and organs.

## Abstract (web summary)

Single-cell and spatial transcriptomic analysis of eight human heart tissues reveals the cellular profiles and tissue architecture of niches including the cardiac conduction system, and a new tool, drug2cell, identifies drug target expression.

## Main

The heart is composed of distinct tissues that contain niches of specialized cell types conferring site-specific functionality. Single-cell RNA sequencing (scRNA-seq) and single-nuclei RNA sequencing (snRNA-seq) offer a powerful, unbiased framework to characterize these cells^2,3^. The addition of spatially resolved transcriptomics allows us to restore structural information lost in single-cell techniques and to gain insight into collective function^4,5^.

The cardiac conduction system (CCS), responsible for the regular and coordinated electrical activation of the heart, contains structures including the sinoatrial and atrioventricular nodes (SAN and AVN, respectively), the atrioventricular bundle (AVB) and the His-Purkinje network, which are each home to cells with distinct electrophysiological properties^6^. We combine targeted dissection and histology to generate full-breadth transcriptomics profiles of human CCS cells. Furthermore, we use spatial transcriptomics to map these cells into their microanatomical locations and discover their niche-partner cells. Inspired by the broad receptor expression profile of pacemaker cells (P cells), we extend the cell–cell interaction database CellPhoneDB^7^ with a new neural–GPCR module. This module highlights synaptic connections, including new insights into neighbouring glial cells and glutamatergic signalling capability.

Off-target activity of non-cardiac therapies on the heart and its conduction system is a major reason for drug development failure and withdrawal^8^. To help address this challenge, we develop a pipeline, drug2cell, which integrates drug–target interactions from the ChEMBL database with user-provided single-cell data to comprehensively evaluate drug-target expression in single cells. Applying this approach to P cells provides mechanistic insight into the chronotropic effects of non-cardiac medications.

Finally, we show that our integrated multiomics approach is capable of niche (that is, cellular tissue microenvironment) discovery. We define an epicardial immune defence system and a ventricular myocardial-stress niche, and infer specific intercellular signalling active within each cellular microenvironment. These new cardiac cellular niches enable us to refine the cellular components that underlie the microanatomy of the human heart.

## Multimodal profiling of the human heart

**Fig. 1:**
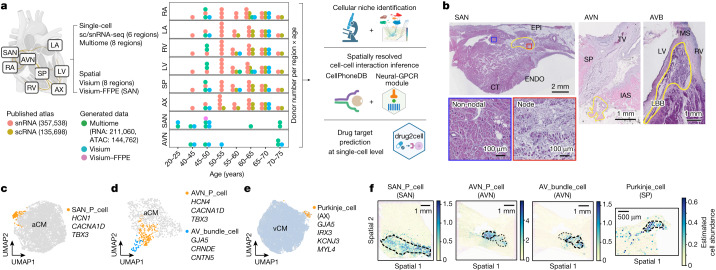
Multimodal profiling of the adult human heart. **a**, Left, overview of study design and analyses. Multiome and Visium spatial transcriptomics data were generated from eight regions (RA, LA, RV, LV, SP, AX, SAN and AVN) of the adult human heart and integrated with a published sc/snRNA-seq atlas dataset^2^. Middle, the dot plot shows the donor numbers by age group (*x* axis) and region (*y* axis). Dot colour represents data modality. The number of cells or nuclei is shown in parentheses. Right, data were used for cellular niche identification, spatially resolved cell–cell interaction analyses and drug-target discovery analysis (drug2cell). **b**, H&E micrographs of the SAN, the AVN and the AVB (yellow bordered). P cells in the nodal tissue (red box) are smaller than neighbouring CMs in non-nodal tissue (blue box) and embedded in dense ECM. The AVB is pictured at its transition to the left bundle branch (LBB). Images are representative of sections from four (SAN), two (AVN) and four (AVB) donors. CT, crista terminalis; ENDO, endocardium; EPI, epicardium; IAS, interatrial septum; MS, membranous septum; TV, tricuspid valve. **c**–**e**, UMAP embedding of gene expression data of SAN aCMs (**c**), AVN aCMs (**d**), and AX and AVN CMs (**e**). Marker genes of CCS cells are shown. **f**, Abundance of CCS cell states in spatial coordinates of SAN, AVN and SP Visium sections as estimated by cell2location. Dashed lines highlight SAN, AVN, AVB and Purkinje cells defined by histology (Extended Data Fig. 2g). Illustrations in **a** were created using BioRender (https://biorender.com). The CellPhoneDB illustration is courtesy of the Wellcome Sanger Institute.

We integrated previously published scRNA-seq and snRNA-seq (sc/snRNA-seq) datasets^2^ with newly generated multiome data (paired snRNA-seq and single nucleus assay for transposase-accessible chromatin using sequencing (snATAC-seq)) and spatial transcriptomics data (10x Genomics). We studied the following eight anatomical regions: the left and right ventricles (LV and RV, respectively), the left and right atria (LA and RA, respectively), the apex (AX), the interventricular septum (SP), the SAN and the AVN (Fig. 1a). In total, our data included samples from 25 donors ranging from 20 to 75 years old (Fig. 1a). All tissue samples were from transplant donors without a history of cardiac disease or arrhythmia (Supplementary Table 1), and hearts contributing to the SAN and AVN regions were from donors with normal conduction parameters confirmed by 12-lead electrocardiograms before donation (Supplementary Table 1).

To capture CCS tissues from the SAN and the AVN regions, we used a two-stage dissection protocol (Supplementary Fig. 1). The presence of CCS structures was confirmed by haematoxylin and eosin (H&E) histology in adjacent sections from the same blocks (Fig. 1b). The SAN forms a discrete subepicardial structure at the junction of the crista terminalis and the RA posterior wall, with P cells smaller and rounder than neighbouring cardiomyocytes (CMs) and surrounded by fibroblasts (FBs) and dense extracellular matrix (ECM) (Fig. 1b and Supplementary Fig. 1a). For the AVN, tissue samples including the triangle of Koch and the basal septum were collected (Fig. 1b and Supplementary Fig. 1b). Subsequently, adjacent sections were taken for spatial transcriptomics analysis. The remaining tissue was sectioned and homogenized to generate nuclei suspensions for multiome analysis.

**Extended Data Fig. 1:**
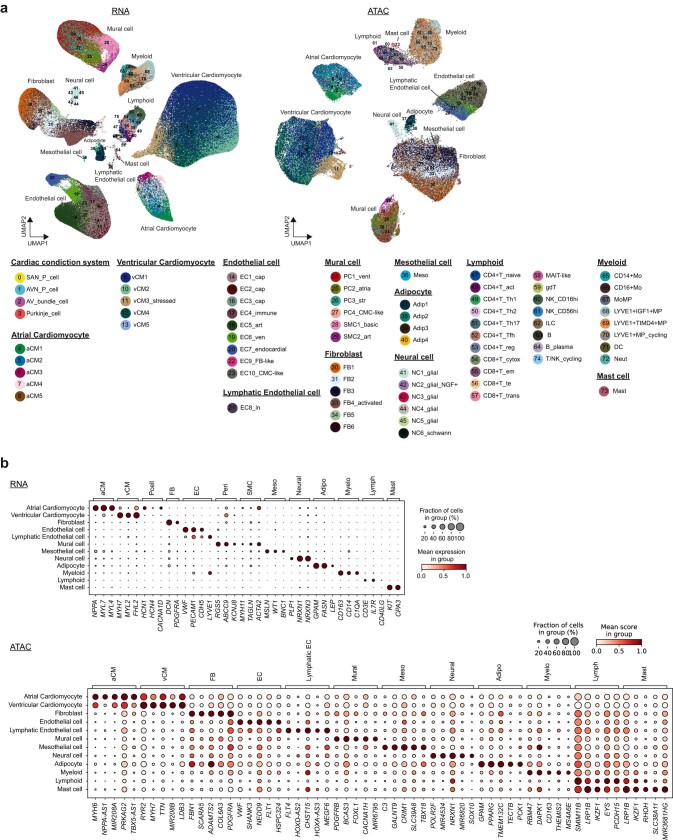
Profiling of cardiac cells. **a**. UMAP representation of from the eight regions showing the fine-grained cell state labels. The data were integrated based on their gene expression (left) or chromatin accessibility (right) with accounting for batch effects and embedded in two-dimensional UMAP space. **b**. Dot plot showing the expressions of curated lineage-specific genes (upper) and the gene scores of differentially accessible genes (lower) in the 12 major cell types.

After sample processing and quality control, 704,296 cells and nuclei (of which 211,060 nuclei were from newly generated multiome data) were retained for gene expression, and 144,762 nuclei were retained for ATAC-seq analyses (Fig. 1a). Integration of gene expression data was performed and accounted for batch variations such as donor, cell or nuclei and the 10x Genomics chemistry version used (Supplementary Fig. 2a) while retaining the regional differences of the atrial (RA and LA), ventricular (RV, LV, SP and AX) and conduction system (SAN and AVN) samples (Supplementary Fig. 2b). Leiden clustering was performed on the neighbourhood graph created using batch-corrected latent variables. The analyses identified 12 coarse-grained cell types annotated using curated lineage-specific gene markers (Extended Data Fig. 1a,b).

**Extended Data Fig. 2:**
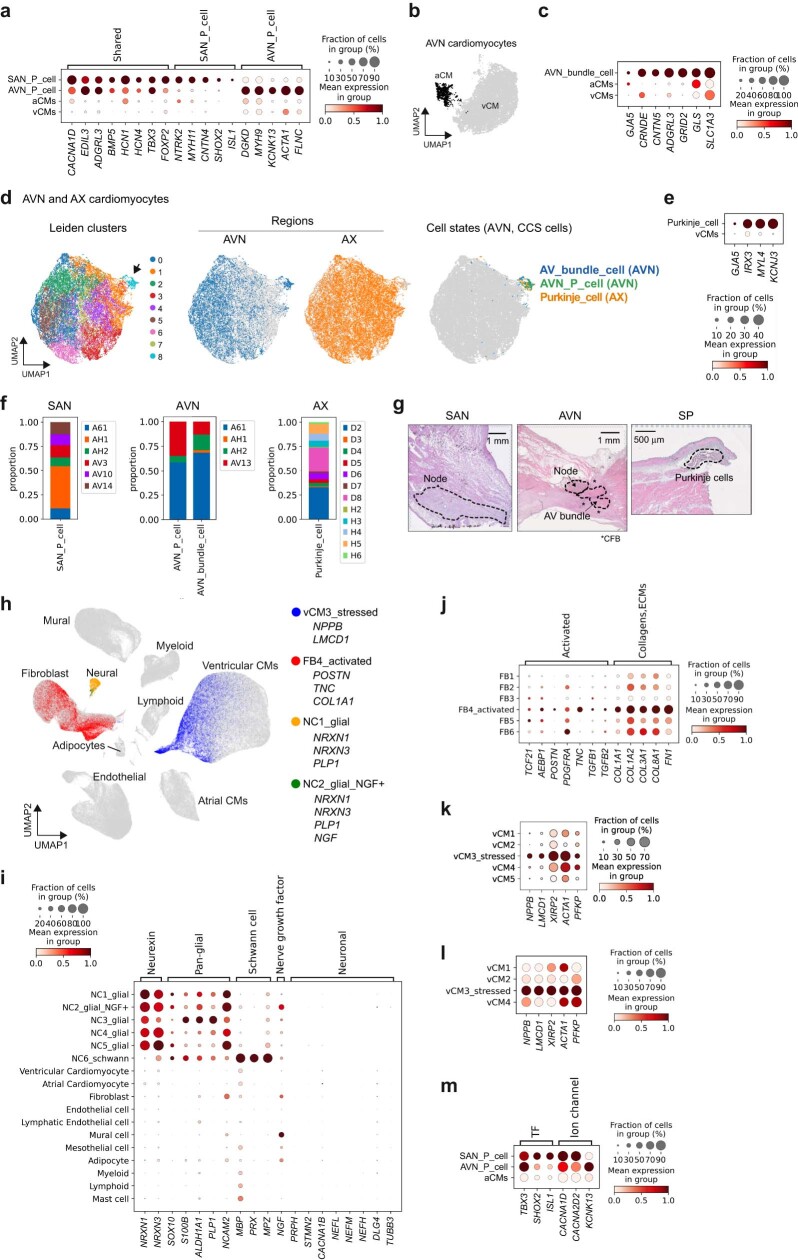
Identification of fine-grained cell states. **a**. Dot plot showing the marker gene expressions of SAN and AVN P cells. **b**. UMAP representation of gene expression data of AVN atrial and ventricle CMs. Dimensional reduction and leiden clustering were repeated for atrial CMs and CCS cell clusters (colored in black) (Fig. 1d). **c**. Dot plot showing the marker gene expressions of AV bundle cells. **d**. UMAP representation of gene expression data of AX and AVN atrial and ventricle CMs. With the labels of leiden clusters, regions, and AVN CCS cells. **e**. Dot plot showing the marker gene expressions of Purkinje cells. **f**. Donor proportions of CCS cell. **g**. H&E morphology of the CCS spatial transcriptomic samples shown in Fig. 1f. Histologically identified CCS regions are in dashed lines. Images representative of sections from four (‘node’ in SAN), two (‘node’ in AVN), and four (‘AV bundle’ in AVN) donors. The ‘Purkinje cell’ structure was identified in this section only. The middle panel shows a section which is oblique to the long axis of the heart which captures the compact AVN at its junction with the origin of the AV bundle. The bundle penetrates into, but not completely through the CFB (central fibrous body, marked by asterisks) in this section. **h**. UMAP embedding of gene expression data from eight cardiac anatomical regions (704,296 cells and nuclei). Highlighted in colour are four non-CCS cell states with a refined annotation compared to the previously published human heart atlas^2^. **i**. Dot plot showing the expressions of glial, schwann, and neuronal cell marker genes in neural cell populations and other cell types. **j**. Dot plot showing the FB activation and ECM gene signature expressions in FB cell states. **k**. Dot plot showing the signature genes of cardiomyocyte stress in vCM cell states. **l**. Dot plot showing the gene scores of the cardiomyocyte stress signature genes in vCM cell states. **m**. Dot plot showing the gene scores of the marker genes of SAN and AVN P cells.

To identify fine-grained cell states of the CCS, SAN region atrial CMs were subclustered, which revealed a clearly separated cluster of P cells (SAN_P_cell) that expressed canonical pacemaker-associated channel genes (*HCN1*, *HCN4* and *CACNA1D*) and the transcription factor (TF) *TBX3* (ref. ^1^) (Fig. 1c and Extended Data Fig. 2a). Subclustering of AVN atrial and ventricular CMs (aCMs and vCMs, respectively) showed two CCS clusters: P cells (AVN_P_cell) and AV bundle cells (AV_bundle_cell) (Fig. 1d and Extended Data Fig. 2b). AV bundle cells formed a distinct cluster defined by an enrichment of *GJA5* (which encodes the gap junction protein Cx40), *CRNDE* and *CNTN5*, which were previously identified as markers of AV bundle cells in the mouse heart^9^ (Fig. 1d and Extended Data Fig. 2c). Purkinje cells (originally termed fibres) are specialized CM-like cells that constitute the distal ramifications of the ventricular conduction system. They play an important part in propagating impulses from the AV bundle branches and their fascicles to the ventricular muscle and are most abundant at the AX^10^. To identify this rare population at single-cell resolution, CMs from AVN samples were integrated and clustered with those from the AX. Cluster 8 (Extended Data Fig. 2d) contained CCS cells from the AVN and a population derived from the AX expressing Purkinje cell marker genes (*GJA5*, *IRX3*, *KCNJ3* and *MYL4*)^9^ (Fig. 1e and Extended Data Fig. 2d,e). All the identified CCS cells were observed in multiple donors (Extended Data Fig. 2f).

To confirm their identity, CCS cell states defined by sc/snRNA-seq data were mapped in spatial transcriptomics data using cell2location, a deconvolution method capable of resolving even rare cell populations^11^. This analysis showed each cell state localized to their expected histologically identified CCS structures (Fig. 1f and Extended Data Fig. 2g).

Other cell states were defined using label transfer with a published dataset^2^ as a reference. Minor revisions of annotation were included. Based on recent studies of the heart and the gut that defined cells expressing *PLP1*, *NRXN1* and *NRXN3* as glial cells^12,13^, we annotated cells expressing pan-glial gene markers and lacking core neuronal genes (Extended Data Fig. 2h,i) as glia (labelled with the ‘_glial’ suffix). FB4 was named as FB4_activated on the basis of increased expression of TGFβ-responsive FB activation signature genes (*POSTN*, *TNC*, *COL1A1*, *COL1A2*, *COL3A1* and *FN1*), which includes genes encoding ECM proteins, compared with other FBs (Extended Data Fig. 2h,j). vCM3 was named vCM3_stressed on the basis of expression of CM stress markers (*NPPB*, *LMCD1*, *XIRP2*, *ACTA1* and *PFKP*)^3,14^ (Extended Data Fig. 2h,k). Myeloid cell states were annotated as described in the Methods (Supplementary Fig. 2c,d). Collectively, we defined 75 cell states (Extended Data Fig. 1a).

## Chromatin landscape of cardiac cells

snATAC-seq data were analysed to understand the chromatin landscape and the genomic regulation of cardiac cell identity. Coarse-grained cell types were clearly separated in the uniform manifold approximation and projection (UMAP), which indicated that each cardiac cell type has a distinct pattern of chromatin accessibility (Extended Data Fig. 1a,b). In the vCM compartment, vCM3_stressed showed a clear separation in the UMAP (Extended Data Fig. 1a), and regions associated with its marker genes were differentially accessible compared with other CMs (Extended Data Fig. 2l). Differential chromatin accessibility analysis comparing CCS cells and other aCMs identified major markers for P cells such as genes encoding ion channel subunits (*CACNA1D* and *CACNA2D2*) and TFs (*ISL1*, *TBX3* and *SHOX2*) (Extended Data Fig. 2m).

**Extended Data Fig. 3:**
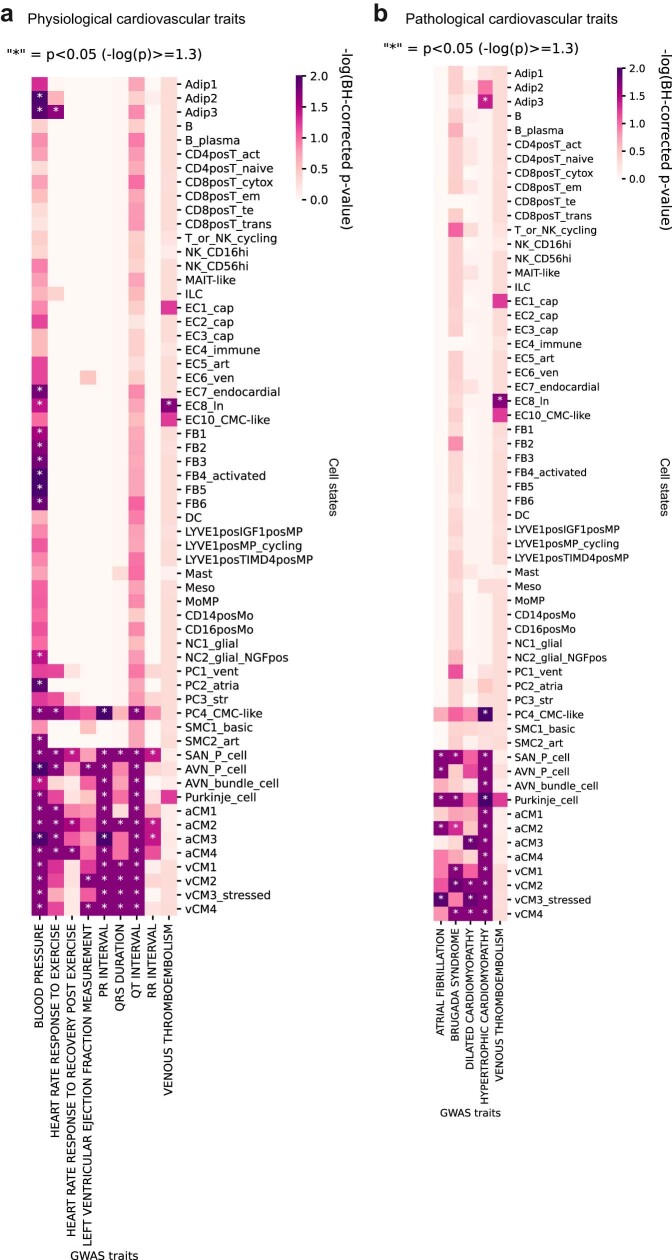
GWAS SNP enrichment analysis. Heatmaps demonstrating enrichment of cell state-specific open chromatin regions for SNPs associated with physiological (**a**) and pathological (**b**) cardiovascular traits. A non-cardiac trait, ‘Venous Thromboembolism’ is a control trait. This was evaluated using a permutation test (**Methods**), in brief: A binary cell type by peak matrix was created indicating peaks open in cell types. The presence of GWAS trait-associated SNPs in peaks was evaluated. The proportion of SNPs in open peaks was calculated (“SNP proportion”). This was compared to a null distribution of SNP proportion generated by 1000 random permutations of the true open peaks. The *p*-value being the proportion of the 1000 random permutations with a greater SNP proportion than the observed SNP proportion. *p*-values were corrected for multiple testing using the Benjamini-Hochberg method.

Common variant single nucleotide polymorphisms (SNPs) identified by genome-wide association studies (GWAS) are frequently located in noncoding regions^15^. We used snATAC-seq data to link cell types with cardiovascular traits by calculating the enrichment of GWAS trait-associated SNPs in their open chromatin regions (Extended Data Fig. 3a). Traits that reflect ventricular physiology (for example, QRS duration, systolic function and QT interval) showed an expected enrichment among vCMs but also in aCMs and CCS cell states, in particular SAN P cells. This result underscored that fact that ventricular function partly depends on atrial function. Conversely, traits that reflect pacemaker activity (RR interval, heart rate response to exercise and its recovery) were limited to P cells and aCMs. For disease-associated SNPs, vCMs were enriched for cardiomyopathy-associated SNPs as expected; however, hypertrophic cardiomyopathy (HCM) SNPs were also enriched in aCMs (Extended Data Fig. 3b). This result suggests that the frequent atrial involvement in HCM might also be due to a primary atrial myopathy and is not merely a secondary phenomenon. The result is also consistent with the finding of atrial dysfunction in patients with HCM carrying pathogenic variants who have not developed ventricular hypertrophy. Conversely, atrial fibrillation SNPs were significantly enriched in CCS cells, aCM2 cells and vCM3_stressed clustered cells. The latter finding emphasizes the interdependence of atrial and ventricular function and supports the strong epidemiological evidence that abnormal ventricular function may drive atrial fibrillation^16^.

## Unbiased discovery of cellular niches

**Fig. 2:**
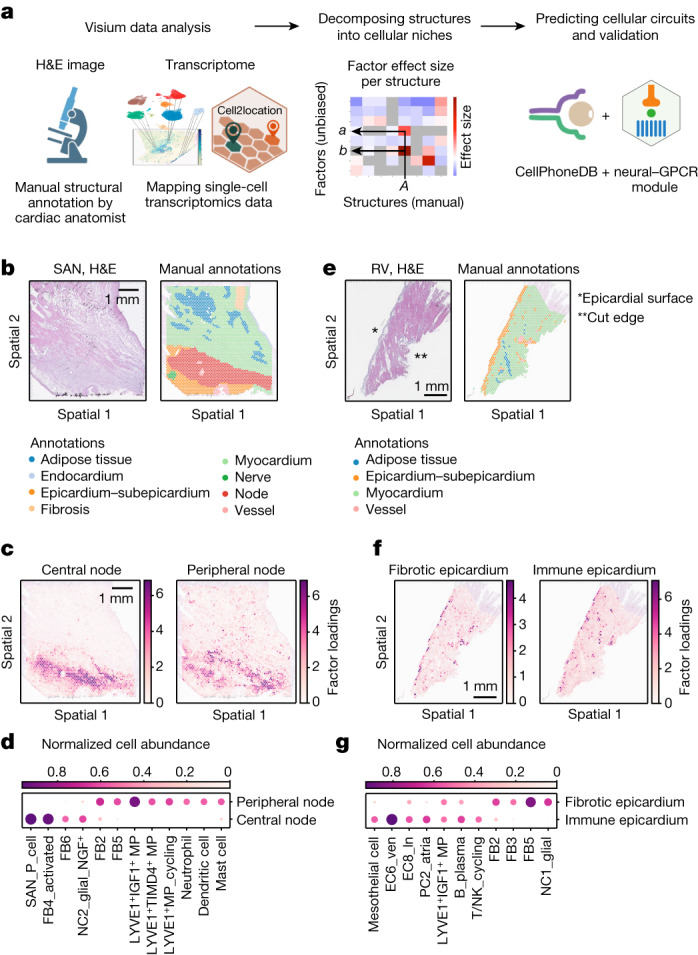
Identification of cellular niches in the adult human heart. **a**, Overview of the spatial data analysis workflow. Visium spots were histologically annotated. Cell states defined by sn/scRNA-seq analysis were mapped to Visium spots using cell2location. NMF was used to decompose manually annotated structures into factors. Spatially resolved analysis of cell–cell interactions was performed using a custom neural–GPCR CellPhoneDB module. **b**–**g**, Cellular microenvironment identification in the SAN (**b**–**d**) and the RV (**e**–**g**). Histological structures were manually annotated on the basis of H&E stainings (representative of four hearts) (**b**,**e**). Factor loadings (estimated abundance of cell state group) of factors identified using cell2location NMF analysis are shown in spatial coordinates (**c**,**f**). Dot plots illustrate cell states with more than 0.4 normalized cell abundance (dot colour and size) in a factor (**d**,**g**). Illustrations in **a** were created using BioRender (https://biorender.com). The cell2location illustration is reproduced with permission from ref. ^11^, Springer Nature America. The CellPhoneDB illustration is courtesy of the Wellcome Sanger Institute.

**Extended Data Fig. 4:**
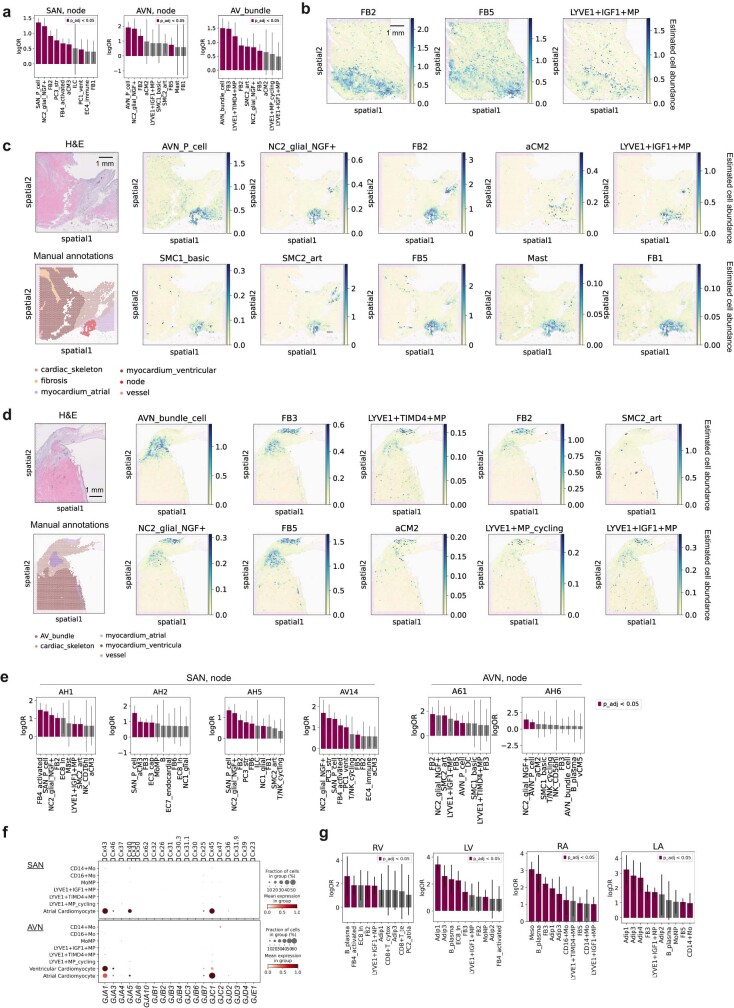
Cell state spatial enrichment in histologically annotated structures. **a**. Cell state enrichment (odds ratio) in the CCS structures: ‘node’ of SAN and AVN, and ‘AV bundle’ of AVN. Visium spot numbers are SAN: 27,108, AVN: 24,026. **b**. Abundance of the cell states (mapped by cell2location) enriched in the ‘node’ of SAN. **c**,**d**. H&E image, manual structural annotations, and abundance of the cell states enriched in the ‘node’ (c) or ‘AV bundle’ (d) of AVN. H&E images are representative of sections from two (‘node’) and four (‘AV bundle’) donors. **e**. Cell state enrichment (odds ratio) in the CCS structures for each donor: ‘node’ of SAN and AVN. Visium spot numbers in the analyses are AH1: 4,243, AH2: 3,802, AH5: 12,781, AV14: 6,282 for SAN and A61: 10,278 and AH6: 2,942 for AVN. **f**. Dot plot showing expression of connexin genes in MP and monocyte cell states from the SAN and AVN regions. **g**. Cell state enrichments (odds ratio) in the ‘epicardium-subepicardium’ of the free wall of the four regions: RV, LV, RA, and LA. Visium spot numbers are RV: 5,039, LV: 9,626, RA: 7,027, and LA: 5,822. Data of cell state spatial enrichment analyses (a,e,g) show log odds ratio with upper and lower 95% confidence interval, and statistically significant enrichments (chi-square test, *p* < 0.05, *p*-values were adjusted for multiple comparisons using the Benjamini-Hochberg method) are shown in magenta-pink.

To understand the cellular composition of microanatomical structures, we mapped the fine-grained cell states defined by sc/snRNA-seq analysis to spatial transcriptomics data using cell2location^11^ (Fig. 2a). In parallel, histological structural annotation based on H&E images was performed by an expert (S.Y.H.). Mapping of expected cell types to the structures was confirmed (Supplementary Fig. 3a–d). In the histologically annotated node of SAN samples, we found enrichment of P cells and other cell states, such as FBs and glial cells expressing NGF (which encodes neuronal growth factor) (NC2_glial_NGF^+^) (Extended Data Fig. 4a,b). Similarly, the annotated node and AV bundle structures of AVN samples from multiple donors included FBs, NC2_glial_NGF^+^ and tissue-resident macrophages (MPs) (LYVE1^+^ MP) together with P cells or AV bundle cells, respectively (Extended Data Fig. 4a,c–e). In contrast to results reported for mouse AVN^17^, our data did not highlight the expression of connexin 43 (encoded by *GJA1*) or other connexins in MPs and monocytes of the SAN and AVN (Extended Data Fig. 4f). In the epicardium of all four cardiac chambers, we detected enrichment of expected cell states (mesothelial cells, FBs, lymphatic endothelial cells (EC8_ln) and adipocytes), but also plasma B cells (B_plasma) and LYVE1^+^IGF1^+^ MPs (Extended Data Fig. 4g).

**Extended Data Fig. 5:**
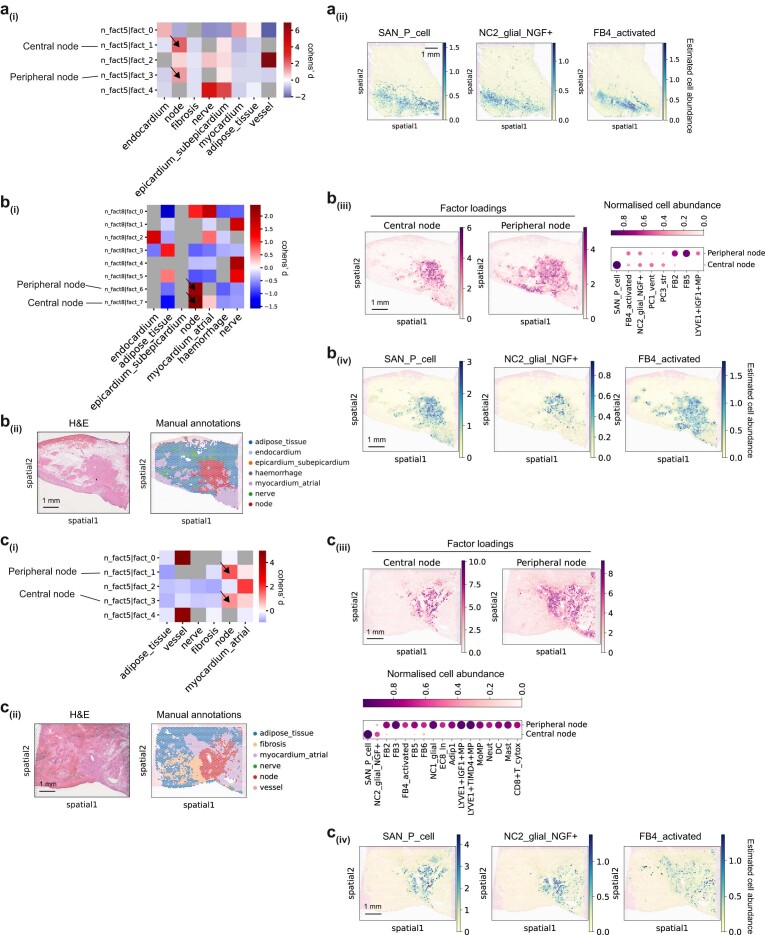
Cellular niche identification in the node. **a**_**(i)**_, **b**_**(i)**_, **c**_**(i)**_. Selected factors which had high effect size in the ‘node’ of SAN visium sections (as indicated by the arrows): donor AH1 (section HCAHeartST10659160) (a_(i)_), donor AV14 (section HCAHeartST13228105) (b_(i)_), and donor AH5 (section HCAHeartST13233996) (c_(i)_). The factor names were assigned based on Fig. 2c, Extended Data Fig. 5b_(iii)_, and Extended Data Fig. 5c_(iii)_. **a**_**(ii)**_, **b**, **c**. Cellular microenvironment identification in the SAN. Manual structural annotations were performed based on the H&E images (b_(ii)_, c_(ii)_). Factors from cell2location NMF analysis which showed high similarity (Cohen’s d) with the ‘node’ structure were selected (as described in Methods). Factor loadings across locations (estimated abundance of cell state group) are shown in spatial coordinates for the selected factors (b_(iii)_, c_(iii)_). The accompanying dot plot illustrates cell states with more than 0.4 normalised cell abundance (visualised by dot size and colour) in the selected factors (b_(iii)_, c_(iii)_). Estimated abundance of representative cell states in the central node of SAN sections (a_(ii)_, b_(iv)_, c_(iv)_). Images of b_(ii)_ and c_(ii)_ are representative of sections from four donors.

To identify cellular niches in an unbiased manner and to decompose the H&E-discernible structures, we performed non-negative matrix factorization (NMF) on the cell2location spot-by-cell output and defined factors of co-occurring cell states^11^. We assessed the similarity between the NMF-identified factors and the manually annotated structures by calculating an effect size of the spot factor loadings in a given structure compared with other areas (Fig. 2a and Supplementary Fig. 4). This analysis confirmed that NMF analysis can define multiple distinct cellular niches within histologically defined structures. For example, the node structure in SAN sections from three donors separated into central and peripheral regions (Fig. 2b,c and Extended Data Fig. 5a(i),b(i–iii),c(i–iii)). The central region contained P cells, FB4_activated and NC2_glial_NGF^+^ cells, whereas the peripheral region was enriched for tissue-resident LYVE1^+^ MPs and other FBs, including the basal FB2 (Fig. 2d and Extended Data Figs. 5a(ii),b(iii,iv),c(iii,iv) and  6a–d). These data clearly indicate that the human SAN is a compartmentalized structure that consists of a central region with the functionally important P cells embedded among activated FBs and glial cells, and surrounded by a peripheral region of immune and other FB populations. This two-layered structure of the SAN may contribute to insulating the P cells to optimize the source–sink relationship and maintain sinus node function^18^. In the node and bundle structures of the AVN region, we observed the enrichment of P cells and NC2_glial_NGF^+^ cells together with FBs and LYVE1^+^ MPs. However, unlike the SAN, there was no compartmentalized cell arrangement, and FB4_activated cells were not abundant (Extended Data Fig. 4a–d).

**Extended Data Fig. 6:**
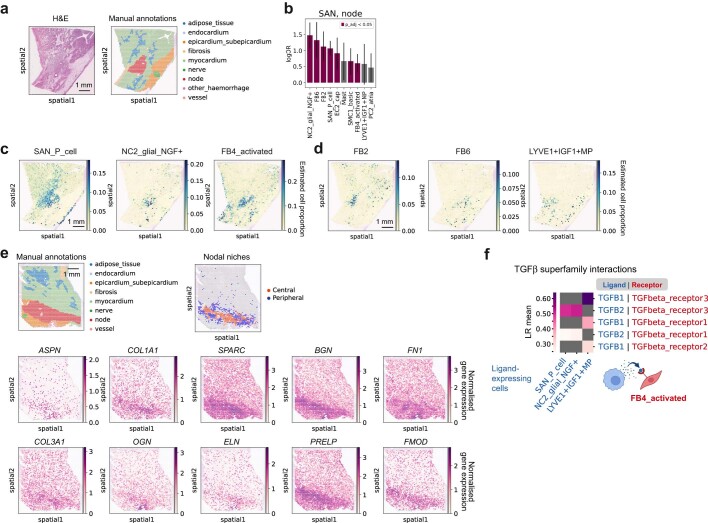
Cellular localisations in the node. **a**–**d**. Analyses on Visium-FFPE slides of the SAN region: H&E image and manual structural annotations (representative of three independent replicates)(a), cell state enrichments in the ‘node’ of SAN (b), and the proportions of the cell states (mapped by cell2location) enriched in the ‘node’ (c,d). Data in (b) show log odds ratio with upper and lower 95% confidence interval, and statistically significant enrichments (chi-square test, *p* < 0.05, *p*-values were adjusted for multiple comparisons using the Benjamini-Hochberg method) are shown in magenta-pink. Visium spot number used in (b) is 11,312. **e**. Expression of extracellular cellular matrix component genes which were differentially expressed in the central nodal niche compared with the peripheral niche (two-sided t-test, *p* < 0.05, log_2_FC>0.5, *p*-values were adjusted for multiple comparisons using the Benjamini-Hochberg method). Each niche was defined based on the NMF analysis and the corresponding spots were selected by thresholding the factor loadings (top 10% of all spots). **f**. Inferred spatial cell-cell interactions involving TGFβ superfamily member ligands in the node, signalling to cognate receptors in the FB4_activated cells (LR mean>0.5). LR mean (mean expressions of the interacting ligand-receptor partners). Illustrations in **f** were created using BioRender (https://biorender.com).

Spatial transcriptomics data showed enrichment of genes that encode ECM proteins and metalloproteinases in the SAN (Extended Data Fig. 6e). CellPhoneDB analysis^7^ of the cells predicted TGFβ ligand–receptor interactions from cells expressing the ligand genes *TGFB1* (LYVE1^+^IGF^+^ MPs) and *TGFB2* (SAN_P_cell and NC2_glial_NGF^+^) to FB4_activated cells expressing the receptor genes *TGFBR1* and *TGFBR3* (Extended Data Fig. 6f). This result suggests that cells from both central and peripheral regions contribute to ECM formation.

**Extended Data Fig. 7:**
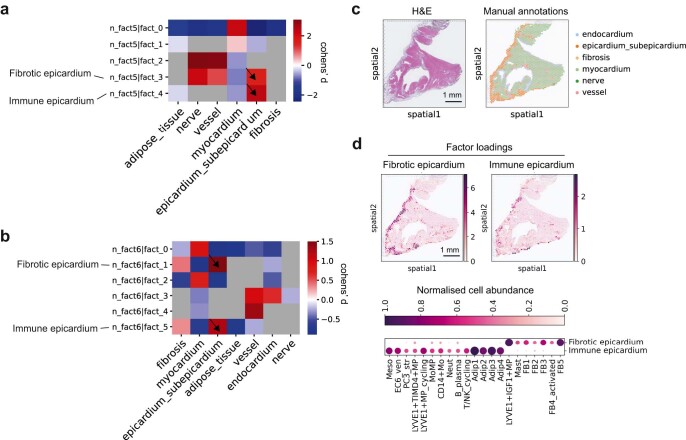
Epicardial cellular niches. **a**. Selected factors (n_fact = 5) which had high effect size in the ‘epicardium-subepicardium’ of RV. The factor names were assigned based on Fig. 2e. **b**. Selected factors (n_fact = 6) which had high effect size in the ‘epicardium-subepicardium’ of LA. The factor names were assigned based on Extended Data Fig. 7d. **c**. Manual structural annotations based on the H&E image. Image representative of sections from four donors. **d**. The factor loadings across locations (estimated abundance of cell state group) are shown in spatial coordinates for the selected factors in (b). The accompanying dot plot illustrates cell states with more than 0.4 normalised cell abundance (visualised by dot size and colour) in the selected factors.

In ventricular and atrial free walls, we assessed the similarities of NMF-identified factors compared with the manually annotated epicardium–subepicardium structure (comprising the epicardial mesothelium monolayer and subepicardial fibrosis) (Extended Data Fig. 7a,b). This analysis decomposed the epicardium–subepicardium structure into a distinct immune epicardium niche (enriched for lymphatic endothelial and immune cells) and a fibrotic epicardium niche (consisting of multiple FB cell states) (Fig. 2e,f and Extended Data Fig. 7c,d). Plasma B cells were present in both epicardial niches, although at a higher proportion in the immune niche (Fig. 2g and Extended Data Fig. 7d).

## Ion channel profile of CCS cells

**Fig. 3:**
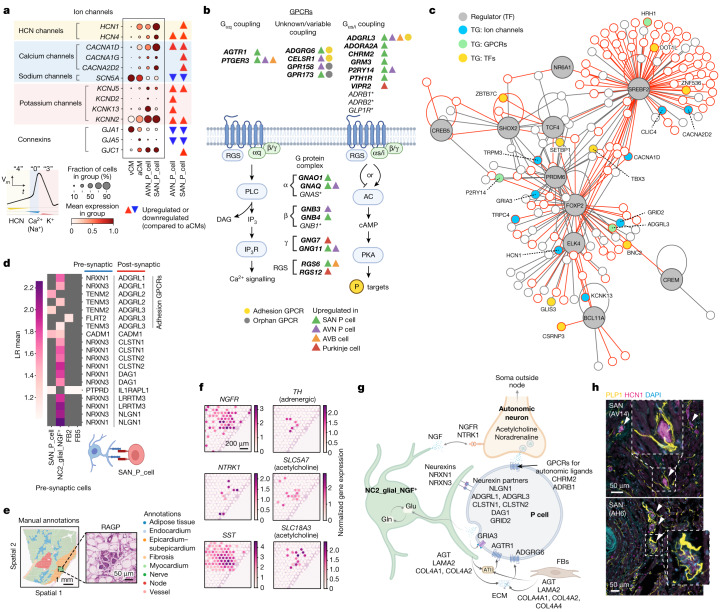
Human P cell profiles and nodal niche interactions. **a**, Top, expression of genes encoding ion channel subunits in P cells. Triangles indicate differential expression. A typical pacemaker action potential is illustrated on the bottom left, with diastolic depolarization (phase 4, yellow), upstroke (phase 0, blue) and repolarization (phase 3, red). Principal currents are depicted with corresponding channel subunit expressions. **b**, Schematic representation of GPCRs and G protein signalling in CCS cells. Differentially expressed genes (versus other aCMs) are in bold. Asterisks mark genes expressed in >10% of P cells that are not differentially expressed (Extended Data Fig. 8e,f). **c**. Predicted TF network governing P cell identity. TFs (grey) and their predicted target genes (TGs) are displayed. Interactions inferred from ATAC-seq analysis are highlighted in red. For a complete list of TGs, see Supplementary Table 5. **d**, Inferred cell–cell (trans-synaptic) interactions between nodal cell states, with ‘receiver’ cells (post-synaptic SAN_P_cells) in red and ‘sender’ (pre-synaptic) cells in blue. LR mean, mean expression levels of the interacting ligand–receptor partners. **e**, Histological annotations of a SAN Visium section (FFPE) and H&E image of a RAGP. Representative of three hearts. **f**, Expression of neural genes in the RAGP (Visium–FFPE). **g**, Schematic representation of interactions in the nodal niche. P cells interact with ECM proteins (secreted by FBs) and NC2_glia_NGF^+^ cells, forming synapse-like connections through neurexins. NC2_glia_NGF^+^ cells support glutamatergic signalling and release NGF, which interacts with receptors on autonomic neurons. P cells express receptors for autonomic neurotransmitters, glutamate and angiotensin II (ATII), which is produced locally by NC2_glial_NGF^+^ cells and FBs. **h**, Immunofluorescence staining of the SAN with anti-HCN1 (pink) and anti-PLP1 (yellow) antibodies and nuclei (DAPI, blue). Arrowheads indicate colocalization of HCN1 and PLP1. Images from two independent donors (AV14 and AH6) are shown. Illustrations in **a**,**b**,**d**,**g** were created using BioRender (https://biorender.com).

**Extended Data Fig. 8:**
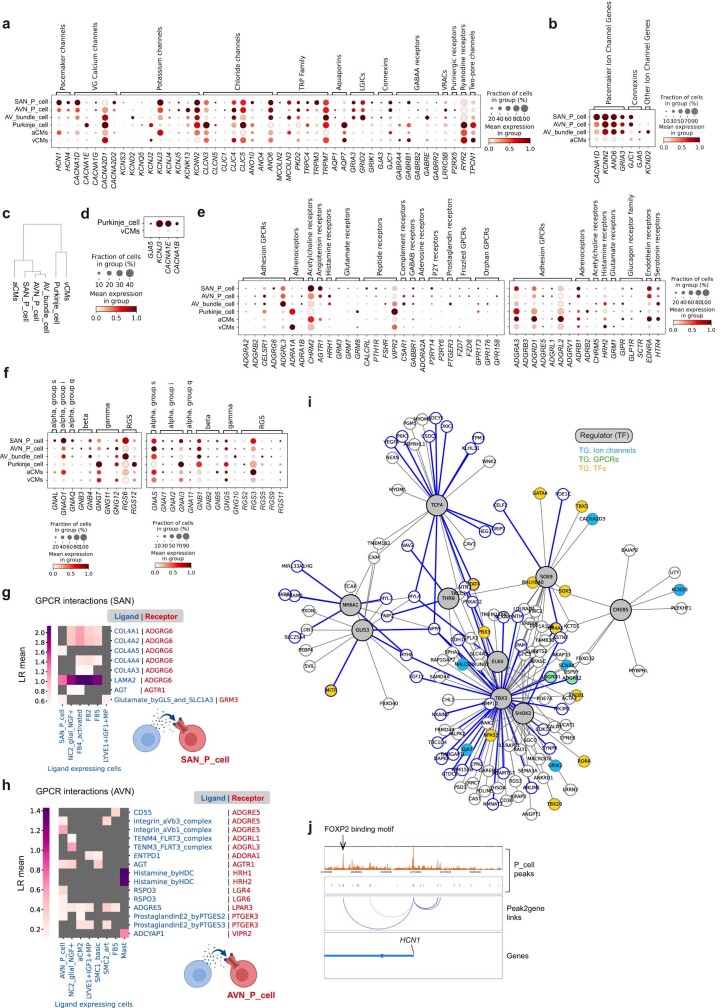
Characterisation of human pacemaker cells and their niche. **a**. Dot plot shows the expression of the DEGs (log_2_FC>0, *p* < 0.05) encoding ion channels in any of the CCS cell states compared with the other aCMs. **b**. Dot plot shows the expression of P cell ion channel genes, genes encoding Connexins 45 (*GJC1*) and 40 (*GJA5*), and the potassium channel gene *KCND2*. **c**. Dendrogram comparing overall gene expression profile of working (aCMs, vCMs) and CCS cell states. **d**. Dot plot shows ion channel genes highly expressed in Purkinje cells compared to other vCMs. **e**,**f**. Dot plot shows the expression of the DEGs (log_2_FC>0, *p* < 0.05) encoding GPCRs (e) or G-protein complexes and RGS (f) in any of the CCS cell states compared with the other aCMs (left). Dot plot shows the genes expressed (>10%) in any of the CCS cell states (right). RGS (regulators of G-protein signalling). **g**,**h**. Inferred spatial cell-cell interactions of GPCRs (CellPhoneDB with neural-GPCR module) in the central node of SAN (g) or the ‘node’ structure of AVN (h), with P cell as the receiver. LR mean (mean expressions of the interacting ligand-receptor partners). **i**. Analysis of TF repressor network in P cells using pySCENIC (**Methods**). TFs (grey) and their predicted target genes (TGs) are displayed. Interactions inferred from snATAC-seq analysis are highlighted in blue. TG colours represent class: GPCRs (green), ion channels (blue), or TFs (yellow). For a complete list of TG see Supplementary Table 6. **j**. Peak-to-gene linkage plot of *HCN1* (ArchR). One of the peaks linked to *HCN1* has FOXP2 binding motif as indicated. DEG testing (a,e,f) was performed with two-sided t-test. *p*-values were adjusted for multiple comparisons using the Benjamini-Hochberg method (Supp. Table 2). Illustrations in **g,h** were created using BioRender (https://biorender.com).

Differential ion channel expression profiles (relative to non-CCS aCMs) highlighted the electrophysiological specialization of the CCS cell states: SAN and AVN P cells, AV bundle cells and Purkinje cells (Fig 3a, Extended Data Fig. 8a and Supplementary Table 2). In contrast to other CMs, the P cell action potential upstroke is mediated by calcium rather than sodium influx^6^. Accordingly, both the SAN and AVN P cells showed downregulation of the sodium channel gene *SCN5A* and upregulation of the L-type calcium channel gene *CACNA1D* (Fig. 3a), the mutation of which causes sinoatrial node dysfunction and bradycardia^19^. Two classical hyperpolarization-activated cyclic nucleotide-gated (HCN) pacemaker channel genes (*HCN1* and *HCN4*) were enriched in P cells from both nodes (Fig. 3a, phase 4). *CACNA1G*, which encodes the T-type calcium channel α-subunit (Ca_V_3.1) and is crucial in pacemaking in rodents^6^, was highly expressed in SAN P cells only. Moreover, the expression of *CACNA1G* was lower than for *CACNA1D*, which highlights the importance of the latter in human pacemaking (Fig. 3a, phase 0). Several ion channels (*CACNA1D*, *HCN1*, *KCNJ3*, *TRPM3* and *CLIC5*) seemed to have cross-species importance because they were also upregulated in mouse SAN P cells (relative to working atrial myocytes) (Supplementary Fig. 5a).

Potassium channel currents contribute to membrane repolarization, which in turn regulates the firing rate of P cells (Fig. 3a, phase 3). *KCNJ5*, a G protein-coupled inwardly rectifying potassium (GIRK) channel subunit was upregulated in both P cell types (Fig. 3a). GIRK channels are directly activated by the βγ-subunit of various GPCRs to modulate cell excitability^6^, which highlights the specialization of P cells to respond to physiological stimuli. Among other potassium channels, *KCNN2* was highly expressed in both P cell types, whereas *KCNK13* was specific to AVN P cells (Extended Data Fig. 8a). The former is activated by intracellular calcium whereas the latter is activated by a range of stimuli, including arachidonic acid and purine receptor agonism^6^.

Various ion channel genes not traditionally associated with cardiac pacemaking were highly expressed in P cells, including several members of the transient receptor potential (TRP) family of nonselective cation channels (*TRPC4*, *TRPM3*, *TRPM7*, *PKD2*, *MCOLN2* and *MCOLN3*). Notably, *TRPM3* was strongly expressed in SAN P cells (Extended Data Fig. 8a). *ANO6*, which encodes a calcium-activated chloride channel, was upregulated in SAN and AVN P cells and in AV bundle cells (Extended Data Fig. 8a). The role of these ion channels in CCS cell function requires further investigation.

Although there were similarities in the ion channel expression profiles of AV bundle cells and P cells (*CACNA1D*, *KCNN2*, *ANO6* and *GRIA3*), bundle cells were marked by the expression of the high-conductance gap junction subunit Cx40 (Extended Data Fig. 8b). *KCND2*, a gene associated with nocturnal atrial fibrillation^20^ and Brugada syndrome, was also specifically expressed in AV bundle cells. Purkinje cells showed a gene expression profile closest to vCMs (Extended Data Fig. 8c). However, the GIRK channel subunit *KCNJ3* and two classically neuron-associated calcium channel subunits (*CACNA1E* and *CACNA1B*) were more highly expressed in Purkinje cells than in vCMs (Extended Data Fig. 8d). Consistent with a fast-conducting phenotype, AV bundle and Purkinje cells were enriched for *GJA5* expression, whereas P cells expressed the ultra-low conductance gap junction subunit *GJC1* (which encodes Cx45)^6^ (Extended Data Fig. 8c).

Altogether, these data provide a highly specific map of genes and cells of the conduction system and may guide future functional studies defining cell-specific druggable targets.

## GPCR profile of CCS cells

Heart rate is tightly regulated by various neurohumoral factors, primarily through GPCR signalling^21^. CCS cells exhibited a wide range of GPCRs, G protein subunits and second-messenger machinery (Extended Data Fig. 8e,f). Within the group of receptors were the classical acetylcholine muscarinic (M_2_) (encoded by *CHRM2*) and catecholamine β (encoded by *ADRB1*) receptors, which are responsible for mediating the major heart-rate-modulating effects of the autonomic nervous system (ANS)^22^. In addition, we found receptors for several neurohumoral ligands in P cells, including angiotensin II (encoded by *AGTR1*), calcitonin gene-related peptide (encoded by *CALCRL*), glucagon-like peptide 1 (encoded b*y* *GLP1R*), parathyroid hormone (encoded by *PTH1R*) and vasoactive intestinal peptide (encoded by *VIPR2*). The adhesion GPCR family, which are activated by the binding of their extracellular domain to ligands on neighbouring cells or within the ECM and are involved in neuronal cell migration and synaptogenesis^23^, were highly represented within CCS cells (*ADGRL3*, *ADGRG6* and *CELSR1*) (Fig. 3b, Extended Data Fig. 8e).

To enable a comprehensive analysis of GPCR interactions in the node, we supplemented the CellPhoneDB database^7^ with a custom module of GPCR and trans-synaptic adhesion molecule interactions, adding more than 1,000 new ligand–receptor pairs (Supplementary Tables 3 and 4). Spatially resolved CellPhoneDB analysis showed multiple interactions through adhesion GPCRs expressed in SAN P cells. ECM proteins, including laminin (encoded by *LAMA2*) expressed in NC2_glial_NGF^+^ cells and FBs interacted with the receptor encoded by *ADGRG6* in SAN P cells (Extended Data Fig. 8g). Interaction of angiotensinogen (encoded by *AGT*), the precursor peptide for the ligand angiotensin II, and its receptor encoded by *AGTR1* was predicted in the nodes of both the SAN and the AVN (Extended Data Fig. 8g,h). Spatial transcriptomics data confirmed *AGT* expression in the manually annotated node structure (Supplementary Fig. 5b). This result is consistent with the identification of local renin–angiotensin circuits and a previous study^24^ showing enriched angiotensinogen expression in the human CCS. These GPCR repertoires support CCS cell responsiveness to an array of neurohumoral factors and interactivity with ECM components and cells in their niche.

## Gene regulatory networks in P cells

To reveal gene regulatory networks that influence P cell gene expression profiles, we constructed both activator and repressor networks using multiome data (Fig. 3c, Extended Data Fig. 8i, Supplementary Fig. 5c,d and Supplementary Tables 5 and 6). TF and target gene interactions inferred from the snATAC-seq data analysis largely overlapped with the network predicted by gene expression.

Notably, our results indicated that FOXP2, a TF associated with language centre development in the brain, is a key TF targeting major P cell ion channel genes such as *HCN1* and *CACNA1D*, as well as *TBX3* (a repressor of working myocyte gene programmes^1^) (Fig. 3c and Supplementary Table 5). The *FOXP2* and *HCN1* interaction was also observed in the snATAC-seq analysis (Extended Data Fig. 8j). This result may explain why mice carrying one non-functional *Foxp2* allele have lower pulse rates than wild-type littermates^25^. *PRDM6*, which is associated with heart rate recovery during exercise^26^, regulated multiple genes encoding ion channels and GPCR. *CREB5*, which encodes cAMP-responsive element-binding protein 5 and is associated with atrial fibrillation^27^, was also one of the TFs highly expressed in P cells compared with other aCMs.

In the repressor network, *TBX3* was highlighted as a regulator of genes encoding GPCRs and proteins involved in electrophysiological processes, such as *SCN5A* and *GJA1*, and *NPPA* (Extended Data Fig. 8i and Supplementary Table 6). This result is consistent with a TBX3-dependent mechanism that suppresses the working myocyte programme in P cells^1^. *TCF4*, which is associated with the capacity to increase heart rate during exercise^28^, showed repressive interactions with multiple genes that encode sarcomeric proteins (*MYOM1*, *MYOM2* and *MYL4*) (Extended Data Fig. 8i).

These predicted control mechanisms may be useful in the engineering of better models of human P cells in vitro.

## Glutamatergic signalling in the nodes

**Extended Data Fig. 9:**
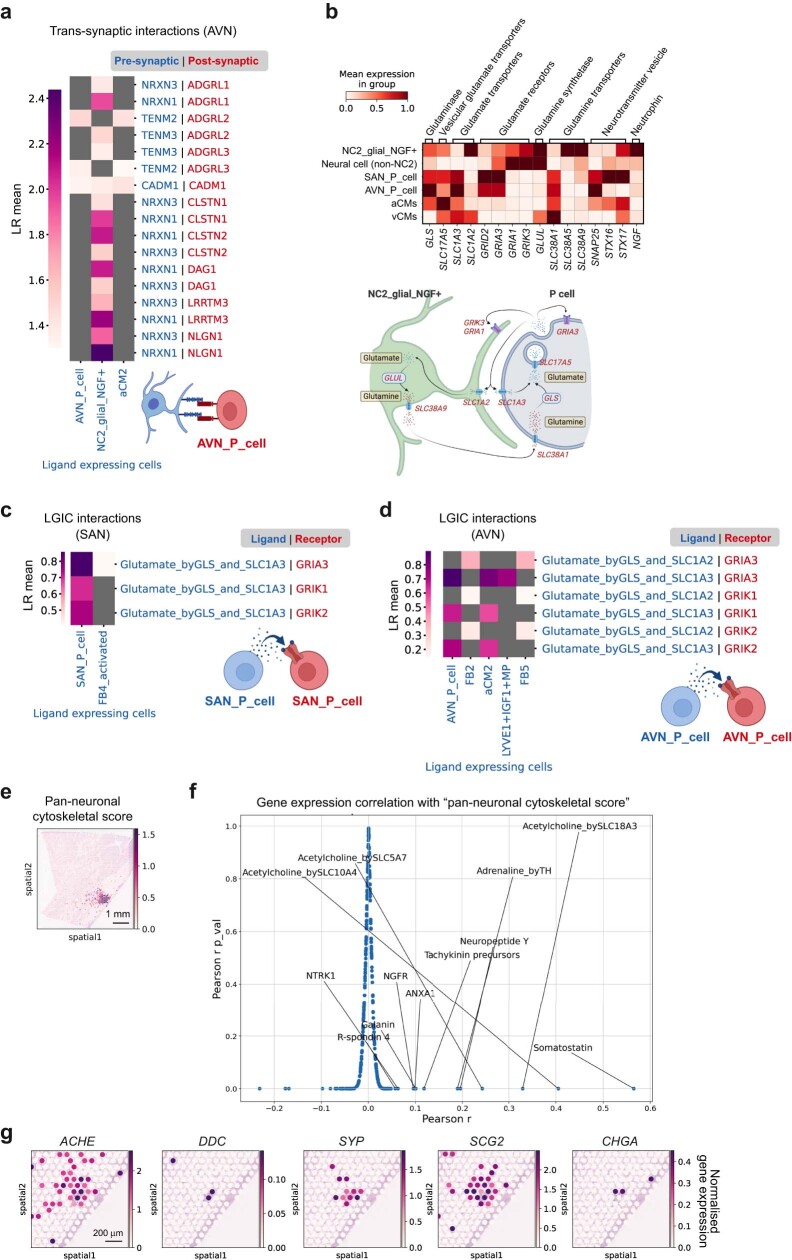
Cell-cell interactions in the node. **a**. Inferred cell-cell trans-synaptic interactions (CellPhoneDB neural-GPCR module) in the AVN, spatially refined by cell states enriched in the ‘node’ structure, with AVN_P_cells as the receiver cells with expression receptors (LR mean>1). LR mean (mean expressions of the interacting ligand-receptor partners). **b**. Mean expression of glutamatergic signalling machinery genes (heatmap, upper). A schematic illustrates paracrine glutamatergic signalling involving P cells and NC2_glial_NGF+ (lower). **c**,**d**. Inferred spatial cell-cell interactions of LGICs in the node of SAN (b) or AVN (c) region, with P cell as the receiver. **e**. Plot of ‘pan-neuronal cytoskeleton score’ (Methods) in spatial coordinates matches RAGP (inset showing RAGP in the associated H&E image in Fig. 3e). **f**. Several GPCR ligands correlate with the pan-neuronal nerve cytoskeleton score. Correlation (Pearson’s r) of individual ligand genes with the pan-neuronal nerve cytoskeleton score (Methods). Ligand genes with *p* value < 0.05 and Pearson’s r > 0.1 are labelled. *p*-values were corrected for multiple testing using the Benjamini-Hochberg method. **g**. Plots of significantly correlated ligand genes in spatial coordinates. Illustrations in **a-d **were created using BioRender (https://biorender.com).

In the histological node structure of both the SAN and the AVN, we observed enrichment and colocalization of P cells with NC2_glial_NGF^+^ cells (Fig. 2d and Extended Data Figs. 4a and 5). This glial population expresses neurexins (*NRXN1* and *NRXN3*), which form trans-synaptic complexes with cell adhesion molecules^29^. Our neural–GPCR CellPhoneDB module highlighted several such interactions with P cells (*NLGN1*, *ADGRL1*, *ADGRL3*, *DAG1* and *CLSTN1*) (Fig. 3d and Extended Data Fig. 9a).

We also discovered that P cells and NC2_glial_NGF^+^ cells express genes involved in glutamatergic signalling. P cells expressed high levels of the glutamate receptor *GRIA3* (an AMPA-type receptor) and *GRID2* (Extended Data Fig. 9b), which encodes a postsynaptic auxiliary subunit that forms a molecular bridge to the presynaptic membrane^30^. The glutamate transporter *SLC1A3* and synthetic enzyme *GLS*, as well as synaptic vesicle genes such as *SNAP25* and *STX7*, were expressed in both SAN and AVN P cells. Spatially resolved CellPhoneDB analysis of the central nodal niche in the SAN or the node structure of the AVN highlighted P cell-to-P cell glutamatergic signalling through three ionotropic glutamate receptors (*GRIA3*, *GRIK1* and *GRIK2*) in both the SAN and the AVN (Extended Data Fig. 9c,d). Expression of *GRIA3* is consistent with the recent observation in rodents of cardiomyocyte–cardiomyocyte glutamate signalling through *Gria3* (ref. ^31^). Opening of these ionotropic channels increases cardiomyocyte excitability and may directly accelerate P cell firing. NC2_glial_NGF^+^ cells expressed genes involved in the glutamate–glutamine cycle, the means by which astroglia replenish glutamine pools for neighbouring glutamatergic neurons^32^. That is, the glutamate reuptake transporter EAAT2 (encoded by *SLC1A2*), glutamine synthetase (encoded by *GLUL*), which remakes glutamine from glutamate, and the glutamine transporter (encoded by *SLC38A9*), which releases extracellular glutamine (Extended Data Fig. 9b). Conversely, P cells expressed glutamine transporter (encoded by *SLC38A1*) and glutaminase (encoded by *GLS*), which converts glutamine to the active neurotransmitter glutamate. These findings suggest that human P cells express the requisite machinery for glutamatergic signalling and that NC2_glial_NGF^+^ cells may form synapses with CCS cells and have an astrocyte-like support role.

## Innervation of the nodes

The majority of cardiac autonomic neurons reside in extracardiac structures (the paravertebral ganglia and brainstem). However, a number of neurons are native to the heart, forming the intrinsic cardiac nervous system (ICNS)^22^. The ICNS neuronal bodies are concentrated within the subepicardial fat in structures known as ganglionated plexi^33^. Our snRNA-seq data did not contain cells matching the profiles of autonomic neurons, probably because of the rarity of ICNS neurons. However, we identified the right atrial ganglionated plexus (RAGP), which houses the somata of neurons innervating the SAN^33^, in six spatial transcriptomics sections across three donors (Fig. 3e). A score calculated for the expression of pan-neuronal cytoskeletal markers (*PRPH*, *NEFL*, *NEFM* and *NEFH*) mapped to the Visium spots on the RAGP region (Fig. 3e and Extended Data Fig. 9e). Correlating the score with gene expression for the corresponding Visium spots enabled us to reveal a transcriptome-wide profile of a human ganglionated plexi (Extended Data Fig. 9f and Supplementary Table 7). The neuropeptide somatostatin (encoded by *SST*), previously identified as a marker of RAGP neuronal populations in pig^33^, was highly correlated with the pan-neuronal cytoskeletal score. Other significantly correlated genes were the cholinergic markers *SLC18A3*, *SLC5A7*, *CHAT* and *ACHE*, the catecholamine synthetic enzymes *TH* and *DDC* and the sympathetic co-transmitter neuropeptide Y (encoded by *NPY*) (Fig. 3f and Extended Data Fig. 9f,g). The relevant corresponding receptors (adrenergic and cholinergic) were also expressed in P cells. The expression of several markers typical of neuroendocrine cells, such as synaptophysin (encoded by *SYP*), secretogranin II (encoded by *SCG2*), chromogranin A (encoded by *CHGA*) and chromaffin granule amine transporter (encoded by *SLC18A1*), also correlated with the pan-neuronal cytoskeletal score (Supplementary Table 7).

Cardiac sympathetic neuron viability depends on a continuous source of the neurotrophin NGF, with recent work in rats suggesting that CMs are a source^34^. However, in our human dataset, *NGF* was more highly expressed in NC2_glial_NGF^+^ cells than in CMs and other neural cells (Extended Data Fig. 2i). Furthermore, the expression of NGF receptors p75NTR (encoded by *NGFR*) and TrkA (encoded by *NTRK1*) correlated with the pan-neuronal cytoskeletal markers (Fig. 3f and Extended Data Fig. 9f). In porcine RAGP, *NTRK1* expression was found specifically in neurons with axonal projections to the SAN^33^. Thus, nodal NGF signalling from NC2_glial_NGF^+^ cells may promote and maintain innervation from the nearby RAGP.

Overall, we propose a nodal cellular circuit wherein NC2_glial_NGF^+^ cells play a pivotal part. That is, they synaptically interact with P cells, facilitating glutamatergic signalling (which potentially modulates firing rate) and promoting autonomic innervation through NGF signalling (Fig. 3g). Immunofluorescence staining of the node with antibodies targeting markers of glial cells (PLP1) and P cells (HCN1) confirmed the proximity of these two cell types in the node (Fig. 3h). In particular, processes from PLP1-positive glial cells seemed to contact HCN1-positive P cells, thereby providing support for our proposed model.

## Drug targeting at single-cell resolution

**Fig. 4:**
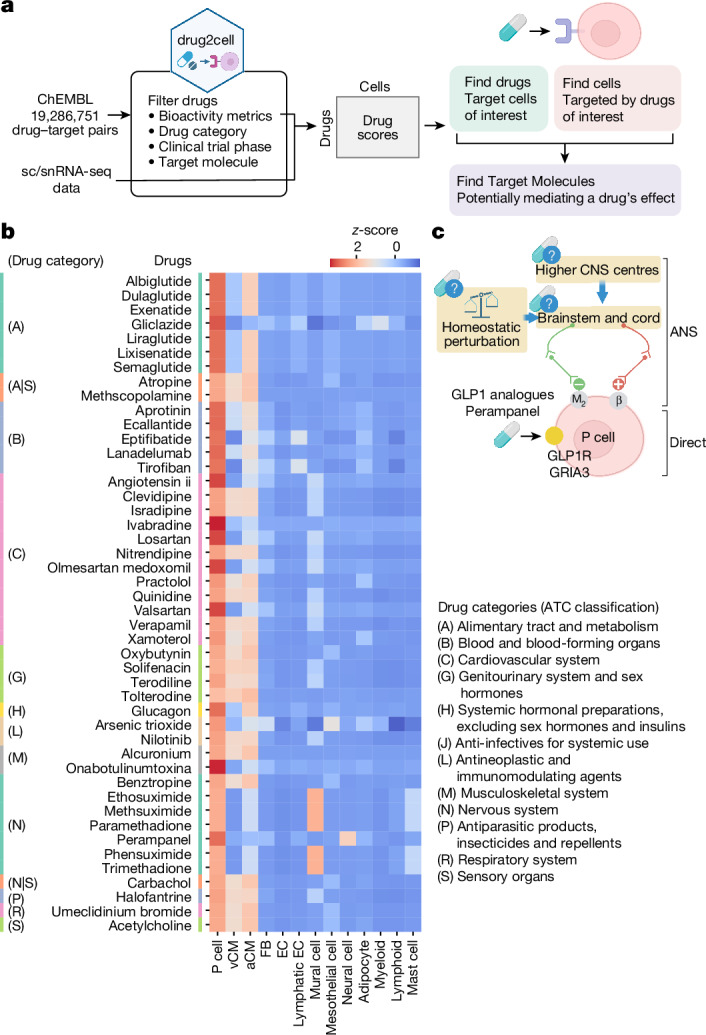
Drug target exploration at the single-cell level. **a**, Schematic of drug2cell analysis. Drugs and their target molecules from the ChEMBL database were queried and filtered on the basis of bioactivity metrics, Anatomical Therapeutic Chemical (ATC) classification, clinical trial phase and classes of molecular targets. Single-cell gene expression data were used to calculate scores and to predict drug interactions for a given cell state. Scores were used to achieve the following aims: (1) find cells targeted by drugs of interest; (2) find drugs targeting cells of interest; and (3) find target molecules expressed in the target cells to infer the effect of a drug. **b**, Heatmap of drug scores that are significantly higher in P cells compared with other cell states (two-sided Wilcoxon test, log_2_(fold change) > 2, *P* value < 0.05, corrected using the Benjamini–Hochberg method). Heatmap colours show the values scaled to *z*-scores for each drug. Categories of drugs are based on the ATC classification. EC, endothelial cell. **c**, Schematic representation of putative cell targets for chronotropic drugs. Single-cell profiling revealed that P cells express genes encoding targets of chronotropic drugs (yellow circle), indicative of ANS-independent mechanisms, such as in the case of the GLP1 analogues and perampanel. β, β-adrenergic receptor; M_2_, muscarinic acetylcholine receptor M2. Illustrations in **a**,**c** were created using BioRender (https://biorender.com).

The cells of the CCS are important targets for chronotropic drugs. Therefore, we sought to leverage our human heart data to map drugs to target these cells. Several single-cell studies and methods have been published that use drug-response transcriptional signatures obtained from cell line experiments and data-mining^35^ to predict drug effects. However, these studies require the development of cell-type-specific in vitro models, which can fail to fully recapitulate the profiles of their in vivo human counterparts. To comprehensively evaluate drug-target expression using single-cell data, we developed a Python package, drug2cell, which uses pairs of drugs and their target molecules from any drug or target database. In this instance, we used the ChEMBL database (https://www.ebi.ac.uk/chembl/) (Fig. 4a). With this streamlined workflow, we applied selected drug–target pairs to single-cell datasets that can be filtered on the basis of quantitative bioactivity metrics, drug categories and clinical trial phases, and target molecule classes. Once drug scores were calculated, it was possible to achieve the following targets: (1) find cells that are targeted by drugs of interest; (2) find drugs that target cells of interest; and (3) find target molecules expressed in the target cells and potentially mediate the effect of the drug (Fig. 4a).

**Extended Data Fig. 10:**
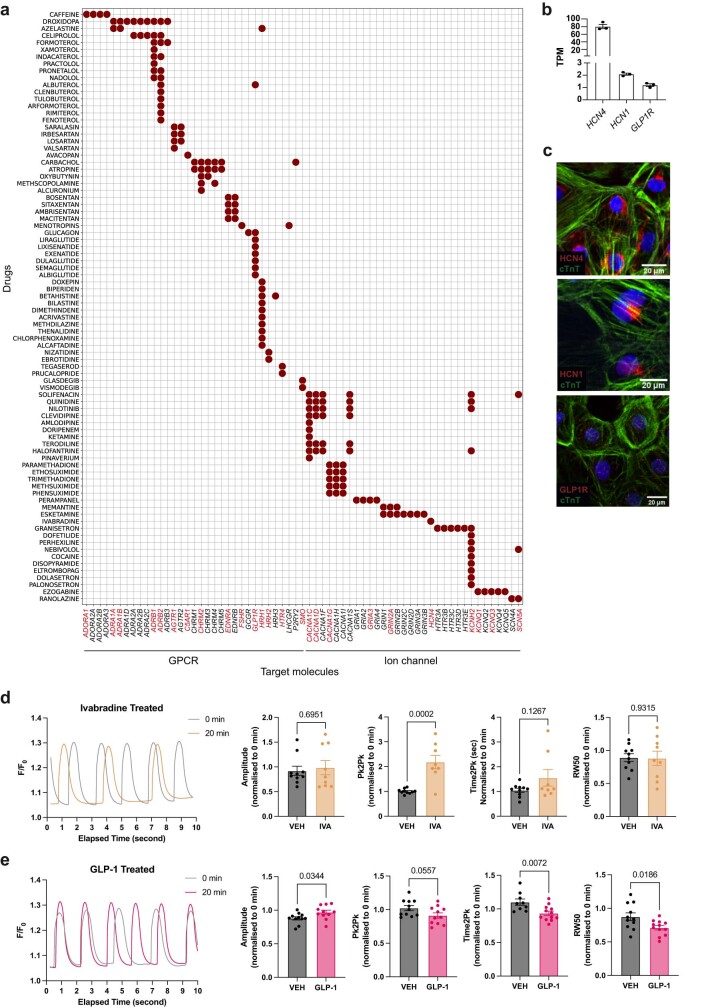
In vitro validation of chronotropic effects of GLP1. **a**. Dotplot shows clinically approved drugs (y-axis) which target GPCRs or Ion channels and had higher scores in P cells compared to other cell states. Genes encoding their molecular targets are shown on the x-axis. Target genes expressed in ≥10% of P cells are highlighted in red. **b**. Bar graphs showing normalised transcript per million (TPM) values obtained from bulk RNA sequencing of hiPSC-CMs for genes encoding for HCN channels and GLP1R. Data shown as mean ± SEM; n = 3 independent experiments. **c**. Representative confocal images of HCN4, HCN1 and GLP1R expression in hiPSC-CMs. Cardiac troponin T (cTnT) was used to visualise CMs and nuclei were stained with DAPI. Images representative of three independent replicates. **d**. Left, representative calcium transient peaks, shown as relative fluorescence intensity (F/F0), detected in hiPSC-CMs before and after 20 min of Ivabradine treatment. Right, bar graphs showing normalised Amplitude, Pk2Pk, Time2Pk and RW50 values. Data shown as mean ± SEM; unpaired two-tailed t-tests; N = 3 independent differentiation batches, n = 9–12 experimental replicates. **e**. Left, representative calcium transient peaks, shown as relative fluorescence intensity (F/F0), detected in hiPSC-CMs before and after 20 min of GLP1 treatment. Right, bar graphs showing normalised Amplitude, Pk2Pk, Time2Pk and RW50 values. Data shown as mean ± SEM; unpaired two-tailed t-tests; N = 3 independent differentiation batches, n = 9–12 experimental replicates.

As an example, we explored clinically approved drugs of all categories (Anatomical Therapeutic Chemical classification) registered in ChEMBL to identify the drugs with the strongest predicted effect on P cells compared with other cardiac cell states (Fig. 4b). This analysis highlighted the group of drugs belonging to the ‘Cardiovascular system’ category, including ivabradine (a HCN1 and HCN4 inhibitor), quinidine (a class I antiarrhythmic agent) and atropine (an anticholinergic agent), which are clinically used drugs with chronotropic effects and are known to act on P cells^36^. To further explore other groups of drugs that potentially affect P cell function, we searched broadly for clinically approved and preclinical bioactive molecules with drug-like properties that target GPCRs or ion channels. As a result, we found drugs that target the GPCRs *ADRB1*, *ADRB2*, *CHRM2* and *GLP1R* and the ion channels *CACNA1C*, *CACNA1D*, *CACNA1G*, *HCN4* and *TRPV1* expressed in P cells (Extended Data Fig. 10a and Supplementary Fig. 6). In addition, we found several drugs that block the angiotensin II receptor (encoded by *AGTR1*), which indicated that these drugs potentially have a direct effect on P cells.

Notably, our analysis identified P-cell-expressed targets for non-cardiac medications with documented chronotropic effects (Fig. 4b and Extended Data Fig. 10a). These included the antidiabetic medication liraglutide (a GLP-1 analogue) and the anti-epileptic medication perampanel (an AMPA receptor inhibitor) (Fig. 4b and Extended Data Fig. 10a). Both drugs are known to alter heart rate, but as their targets were not known to be present in P cells before this study, alternative sites of action (the ANS and CNS, respectively) had been proposed as mediators of these effects^37,38^ (Fig. 4c).

To test the effect of GLP-1, we used human induced pluripotent stem-cell-derived cardiomyocytes (hiPSC-CMs), which exhibit pacemaker properties such as automatic firing^39^. Expression of the pacemaker channel genes *HCN4*, *HCN1* and *GLP1R* was confirmed at the mRNA level and at the protein level by immunofluorescence staining (Extended Data Fig. 10b,c). Live imaging of intracellular calcium transients was performed on cells treated with GLP-1 or ivabradine, a negative chronotropic agent that slows diastolic depolarization by blocking pacemaker (HCN1 and HCN4) channels as a control. Ivabradine treatment decreased spontaneous firing rates of hiPSC-CMs, as shown by an increase in the time between peak maximum (Pk2Pk) (Extended Data Fig. 10d). By contrast, GLP-1 reduced the time between peak threshold and peak maximum (Time2Pk) value, which is indicative of a positive chronotropic effect (Extended Data Fig. 10e). These results are consistent with the documented chronotropic effects observed in patients treated with GLP-1 analogues and suggest that the effect is at least partially mediated by a direct action on P cells (Fig. 4c).

In summary, drug2cell can identify specific cellular targets of bioactive molecules with drug properties based on sc/snRNA-seq data, potentially revealing hidden mechanisms of action and predicting the impact of medicines on specific cell types.

## Immune niche in the epicardium

**Fig. 5:**
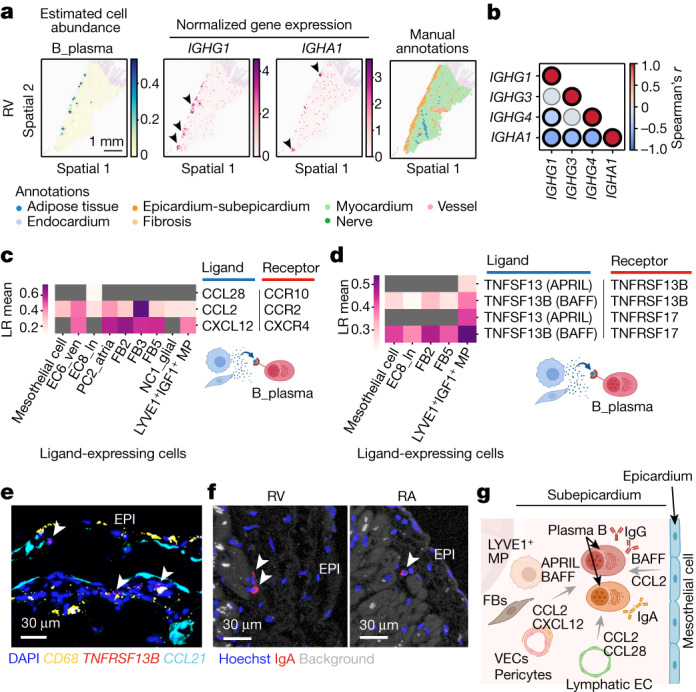
Immune niche in the epicardium. **a**, Images show abundances of plasma B cells (estimated using cell2location), *IGHG1* and *IGHA1* expression, and annotations of histological structures in spatial transcriptomics sections from the RV region. **b**, Correlation between paired *IGH* gene expressions in the epicardium–subepicardium. Colour scale indicates Spearman correlation coefficient and significant correlations (two-sided, *P* value < 0.05, adjusted using the Bonferroni method) are indicated by thick edges. **c**,**d**, Heatmaps summarize the inferred cell–cell interactions mediated by chemokines (**c**) and cytokines (**d**) from cells sending the signals (*x* axis) to the plasma B cells expressing their cognate receptors. LR mean, mean expression levels of the interacting ligand–receptor partners. **e**, Multiplex smFISH (RNAscope) of the LV epicardial region for *TNFRSF13B*, *CCL21* and *CD68*. DAPI was used to stain nuclei. Image representative of three independent replicates. **f**, Immunofluorescence of the epicardial regions (RV and RA) stained with anti-IgA antibody and Hoechst for nuclei. Background signals are white. Image representative of three independent replicates. EPI, epicardium. **g**, Schematic of the immune niche in the epicardium and subepicardium shows plasma B cells, MPs, FBs and ECs. VECs, vascular ECs. Illustrations in **c**,**d**,**g** were created using BioRender (https://biorender.com).

**Extended Data Fig. 11:**
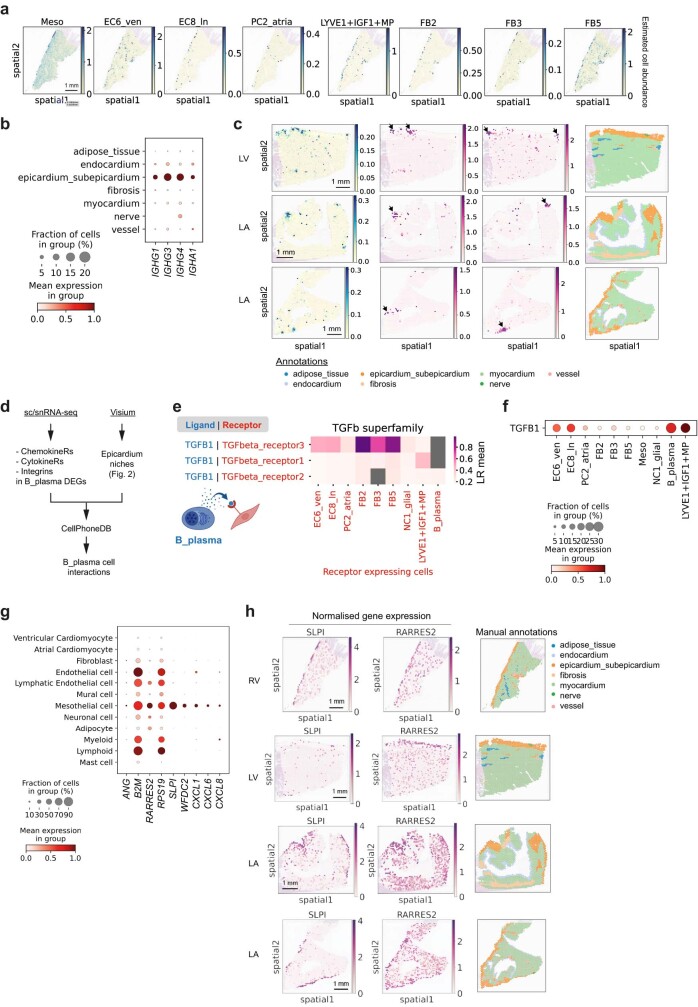
An immune niche in the epicardium. **a**. Abundance of the co-locating cell states (estimated by cell2location) in the ‘epicardium-subepicardium’ structure of RV (Fig. 2f). **b**. Dot plot showing expressions of *IGH* genes significantly enriched in the ‘epicardium-subepicardium’ compared with other manually annotated structures (*p*-value<0.05, log_2_FC>1). **c**. Images show abundances of plasma B cells (estimated using cell2location), mapping of *IGHG1* and *IGHA1* expression, and annotations of histological structures in spatial transcriptomic sections from the indicated anatomical regions. **d**. Workflow of spatial CellPhoneDB analysis focusing on B_plasma cells. **e**. Inferred spatial cell-cell interactions of TGFβ superfamily, spatially refined by niche partner cell states, with plasma B cells as the sender cell expressing ligands. LR mean (mean expressions of the interacting ligand-receptor partners). **f**. Dot plot showing *TGFB1* expression in the cell states localised in the epicardial niches. **g**. Dot plot showing the expressions of ‘antimicrobial humoral response’ genes (Gene Ontology Term, GO:0019730) which were expressed significantly higher in mesothelial cells compared to other cells (*p*-value<0.05, log_2_FC>1). **h**. Expression of antimicrobial response genes, *SLPI* and *RARRES2*, in spatial transcriptomics data. DEG testing (b,g) was performed with two-sided wilcoxon test. *p*-values adjusted for multiple comparisons using the Benjamini-Hochberg method. Illustrations in **e** were created using BioRender (https://biorender.com).

To further understand the immune niche that incorporates epicardial plasma B cells (Fig. 2g and Extended Data Fig. 11a), we localized plasma B cells and the associated genes encoding immunoglobulin heavy chains in the spatial transcriptomics data. Genes encoding the immunoglobulin heavy chains (*IGHG1*, *IGHG3*, *IGHG4* and *IGHA1*) were significantly enriched in the epicardium–subepicardium histological structure (Extended Data Fig. 11b), and the spots expressing *IGHG1* and *IGHA1* showed distinct localization (Fig. 5a,b and Extended Data Fig. 11c). This result suggests that different mechanisms regulate the fate of IgG^+^ and IgA^+^ plasma B cells within the epicardial–subepicardial niche.

To understand the mechanisms that regulate the dynamics of this cellular niche, we performed spatially resolved CellPhoneDB analysis (Extended Data Fig. 11d). Endothelial cells, FBs, mesothelial cells and MPs expressed genes encoding CCL2 and CXCL12 (also known as SDF1), and plasma B cells expressed genes encoding their receptors, CCR2 and CXCR4, respectively (Fig. 5c). This result suggests that plasma B cells may be recruited through a chemokine-dependent mechanism^40^. Our data also pointed towards a CCL28–CCR10 interaction, a specific mechanism for IgA^+^ plasma B cell recruitment^40^, from lymphatic endothelial cells (EC8_ln) to plasma B cells (Fig. 5c). Ligand-encoding genes *TNFSF13B* (which encodes BAFF) and *TNFSF13* (which encodes APRIL) were expressed in MPs, monocytes and FBs, whereas their receptor counterparts *TNFRSF13B* (which encodes TACI) and *TNFRSF17* (which encodes BCMA) were expressed in plasma B cells (Fig. 5d). This result suggests that tumour necrosis factor (TNF) superfamily-dependent signalling contributes to the homeostasis of the niche^41^. Consistent with our CellPhoneDB predictions, multiplex single-molecule fluorescence in situ hybridization (smFISH) analysis confirmed the colocalization of plasma B cells expressing *TNFRSF13B* (which encodes the BAFF receptor) alongside MPs in the epicardium (Fig. 5e). Immunofluorescence staining also confirmed the presence of IgA in the subepicardial region (Fig. 5f). Furthermore, CellPhoneDB predicted interactions between plasma B cells and FBs (FB2, FB3 and FB5), LYVE1^+^IGF1^+^ MPs and endothelial cells (EC6_vec and EC8_ln) through TGFβ1 and TGFβ receptors (Extended Data Fig. 11e). In line with a previous study^42^ showing a key role of plasma B cells in fibrosis, plasma B cells together with LYVE1^+^IGF1^+^ MP and endothelial cells were the major sources of *TGFB1* (Extended Data Fig. 11f).

The manually annotated epicardial–subepicardium structure highly expressed genes for secreted antimicrobial defence molecules, including *SLPI* and *RARRES2* (encoding chemerin)^43^, as well as monocyte/neutrophil-attracting chemokines (*CXCL1*, *CXCL6* and *CXCL8*). This result is consistent with the enrichment of these genes in mesothelial cells (Extended Data Fig. 11g,h), and suggests that they have directly contribute to the immune defensive function of the heart.

In summary, we defined a new epicardial niche in which MP-derived and stromal-cell-derived signals mediate the recruitment and homeostasis of plasma B cells that secrete immunoglobulins. These antibodies, together with other antimicrobial molecules, provide an immune barrier that protects the heart against lung-derived invading pathogens (Fig. 5g), and its dysregulation could contribute to autoimmune mechanisms of cardiac disease^44^.

## Ventricular myocardial-stress niche

**Extended Data Fig. 12:**
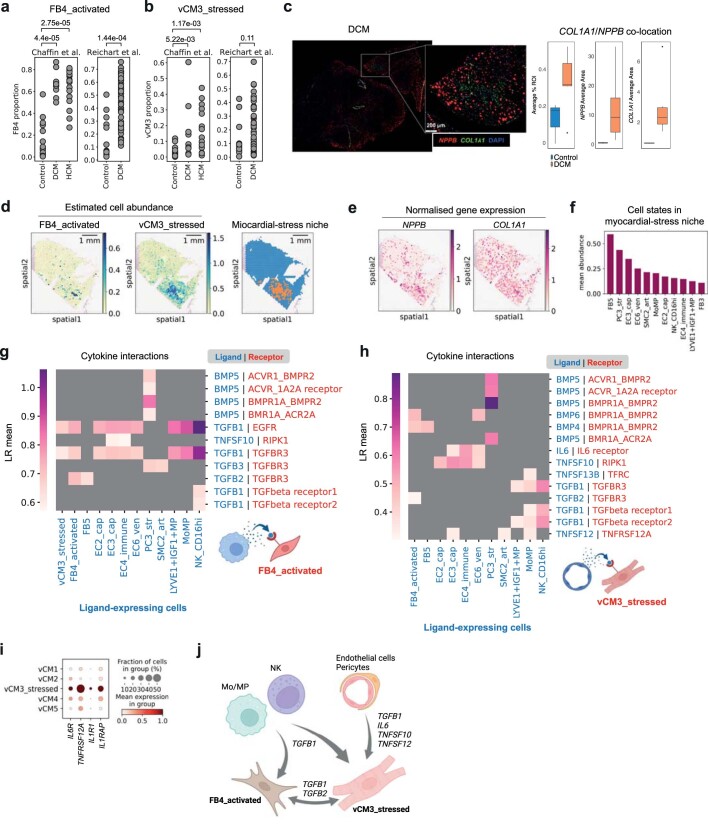
Ventricular myocardial-stress niche. **a**,**b**. Comparisons of FB4_activated proportions amongst FBs (a) and vCM3_stressed amongst vCMs (b) between control and diseased samples in publicly available DCM and HCM datasets^45,46^. Cell state labels were transferred from the dataset in this study using scNym^63^. *p*-values are provided for each comparison (two-sided wilcoxon rank-sum test, *p*-values were adjusted for multiple comparisons using the Benjamini-Hochberg method). **c**. Multiplex single-molecule fluorescence *in situ* hybridization (RNAscope) of left ventricular regions (LV, SP, and AX) for *NPPB* and *COL1A1*. DAPI (blue) was used to stain nuclei. The proportions of the *COL1A1*/*NPPB* co-location and the expressions of each gene in the area of *COL1A1*/*NPPB* co-location were quantified for control (n = 2 donors x 3 regions) and DCM samples (n = 2 donors x 3 regions). For the box plots, the centre line shows the median, the box limits represent the 25th and 75th percentiles, the whiskers show the minimum and maximum values, and the dots represent potential outliers. **d**,**e**. Visium section of SP showing FB4_activated and vCM3_stressed abundances (d), and expression of their markers *NPPB* and *COL1A1* (e). The myocardial-stress niche was defined based on the abundances of FB4_activated and vCM3_stressed (Methods). **f**. Cell states enriched in the myocardial-stress niche, ordered by mean abundance. **g**,**h**. Inferred cell-cell interactions of cytokines in the myocardial-stress niche cell states, with FB4_activated (g) or vCM3_stressed (h) as the receiver. **i**. Dotplot showing inflammatory cytokine receptor expressions in vCM3_stressed and other vCMs of left ventricular regions (LV, SP, and AX). **j**. Myocardial-stress niche in the ventricle. TGFβ superfamily interactions from immune cells and vasculature cells to FB4_activated and vCM3_stressed may cause pathogenic fibrosis. Vasculature cells also express inflammatory cytokines which may directly affect vCMs and lead to adverse cardiac remodelling. Illustrations in **g,h,j** were created using BioRender (https://biorender.com).

From the results above, we noted activated FB cell states with high collagen expression profiles (FB4_activated) and stressed ventricular CMs expressing cardiomyocyte stress signatures (vCM3_stressed) (Extended Data Fig. 2h). By testing their abundance in publicly available datasets of dilated cardiomyopathy (DCM) and HCM^45,46^, we detected expansions of FB4_activated and vCM3_stressed populations in both DCM and HCM samples compared with samples from healthy individuals (Extended Data Fig. 12a,b). To validate this finding, we examined ventricular myocardium samples from patients with DCM using multiplex smFISH. This analysis revealed the colocalization and enrichment of marker genes for FB4_activated (*COL1A1*) and vCM3_stressed (*NPPB*) cells in DCM samples compared with controls (Extended Data Fig. 12c). Spatial transcriptomics analysis of the SP region of donor hearts revealed colocalization of these two cell states with vascular cells and immune cells defining a myocardial stress niche (Extended Data Fig. 12d–f). Spatially refined CellPhoneDB analysis suggested that the TGFβ superfamily contributed to intercellular communications within this microenvironment. Specifically, endothelial cells, smooth muscle cells (SMC2_art), FBs (FB4_activated and FB5) and immune cells (MPs and NK cells) expressed genes encoding TGFβ ligands. By contrast, TGFβ receptors were expressed in both FB4_activated and vCM3_stressed populations (Extended Data Fig. 12g). Also, several endothelial cell populations and SMC2_art cells expressed inflammatory cytokines (*IL6*, *TNFSF10* and *TNFSF12*), which interact with their cognate receptors expressed in vCM3_stressed cells (Extended Data Fig. 12h). This result indicates that there is direct proinflammatory signalling to the cardiomyocytes. In fact, compared with other vCMs, vCM3_stressed cells expressed higher levels of genes encoding receptors for IL-6, TNFSF12 and IL-1 (*IL6R*, *TNFRSF12A*, and *IL1R1* and *IL1RAP*, respectively) (Extended Data Fig. 12i), which are associated with the pathogenesis of heart failure^44^. We speculate that this myocardial-stress niche may be a precursor to adverse cardiac remodelling and pathogenic fibrosis typical of cardiomyopathy (Extended Data Fig. 12j).

## Discussion

Our work sheds new light on the cellular profiles and composition of niches in the human heart. In the CCS, P cells expressed the TF FOXP2 and ion channel genes not traditionally associated with cardiac pacemaking. These include members of the TRP family of non-selective cation channels for which their conductance is regulated by physicochemical stimuli and can modulate cardiomyocyte excitability^47^. *TRPM3*, inhibited by the βγ-subunits of G_i/o_-coupled GPCRs, has been implicated in SAN-associated and AVN-associated GWAS traits^48,49^ and could modulate P cell excitability in a fashion similar to GIRK channels^50^. We also report *ANO6*, which encodes a calcium-activated chloride channel, in human CCS cells. Notably, another member of the same anoctamin family (*ANO1*) is the primary pacemaker channel responsible for peristaltic slow-wave generation in the Cajal cells of the gut^13,51^, and was detected in mouse SAN^52^. We developed drug2cell, a tool to identify drug-target expression at the single-cell level. This tool highlighted P cells as previously unknown cellular targets for drugs with chronotropic side effects (including GLP-1 analogues and perampanel), the mechanisms of which had remained elusive^37^. In the future, applying drug2cell to whole-body cell atlas datasets may improve in silico screening by predicting adverse effects in cell types across all organs.

Through our spatial analyses, we showed that the glial population NC2_glial_NGF^+^ is a niche partner of CCS cells in both nodes and the AV bundle. These cells express key elements required to maintain the glutamine pool and may therefore facilitate cardiac glutamatergic signalling in an astrocyte-like role. Similarly, our analysis highlighted multiple trans-synaptic adhesion interactions suggesting a synapse-like interconnection, as supported by the envelopment of P cells by glial processes. Consistent with the recently suggested cardiomyocyte–neuron signalling circuit^34^, NC2_glial_NGF^+^ cells may promote CCS innervation through the secretion of the neurotrophic factor NGF, the receptors of which (*NGFR* and *NTRK1*) are expressed in the nearby RAGP. Together, these findings indicate an important multifaceted role for NC2_glial_NGF^+^ cells in supporting CCS cells.

The presence of IgA^+^ and IgG^+^ plasma B cells in the epicardium and subepicardium is consistent with a role as a barrier to direct invasion of pathogens such as those infecting the neighbouring lungs. Mesothelial cells may have a key part in the formation of the epicardial immune niche by expressing chemokines (*CCL2*) and pro-survival factors for B cells (*BAFF*) and the immune defensive function by expressing antimicrobial molecules (*SLPI* and *RARRES2*). This role is similar to the immunoregulatory function of mesothelial cells in the peritoneal cavity^53^. Within the myocardium, we discovered a myocardial-stress niche with enrichment of genes encoding inflammatory cytokine receptors (IL-1 and IL-6 receptors) in the stressed CM population (vCM3_stressed). This result implies a mechanism that mediates the susceptibility of the heart to inflammation and remodelling within the myocardial-stress niche^44^.

This framework, which combined multimodal data and integrated knowledge-based and unsupervised microstructural annotations, has the power to drive niche discovery and can be applied to other tissues in health and disease.

## Methods

### Research ethics for donor tissues

All heart tissue samples were obtained from transplant donors after Research Ethics Committee approval and written informed consent from donor families as previously described^2^. The following ethics approvals for donors of additional heart tissue were obtained: D8 and A61 (REC reference 15/EE/0152, East of England Cambridge South Research Ethics Committee); AH1 (DN_A17), AH2 (DN_A18), AH5 (DN_A19) and AH6 (DN_A20) (REC reference 16/LO/1568, London, London Bridge Research Ethics Committee); AV1 (HOPA03), AV3 (HOPB01), AV10 (HOPC05), AV13 (HOPA05) and AV14 (HOPA06) (REC reference 16/NE/0230, North East, Newcastle & North Tyneside Research Ethics Committee). Samples of failing hearts used for validation were obtained under the Research Ethic Committee approval given to the Royal Brompton & Harefield Hospital Cardiovascular Research Centre Tissue Bank (REC reference 19/SC/0257).

### Tissue acquisition and processing

Cardiovascular history was unremarkable for all donors (Supplementary Table 1). Hearts contributing to the SAN and AVN regions were from donors confirmed to be in sinus rhythm with normal conduction parameters by echocardiogram before donation (Supplementary Table 1). Hearts were acquired after circulatory death (D8, A61, AH1, AH2, AH5 and AV3) and after brain death (AV1, AV10, AV13, AV14 and AH6). For donation after circulatory death donors, after confirmation of death there was a mandatory 5-min stand-off before sternotomy. In all cases, the aorta was cross-clamped and cold cardioplegia solution was administered to the aortic root before cardiotomy. Samples AV1, AV3, AV10, AV13 and AV14 were procured in the standard fashion and then immediately preserved with and transported on a hypothermic perfusion machine. Sample AH2 was similarly preserved, but with immediate normothermic perfusion. It then underwent 4 h of normothermic perfusion before samples were taken. For single nuclei sequencing, all donor samples were full-thickness myocardial tissues from the SAN, the AVN, the LA, the RA, the LV, the RV, the SP and the AX. For spatial transcriptomics, ventricle regions, the thicknesses of which were larger than one side of the Visium frame (6.5 mm), were separated into epicardial and endocardial portions. Samples used for single nuclei isolation and spatial transcriptomics were flash-frozen or frozen in OCT and stored at −80 °C, or formalin-fixed and subsequently embedded in paraffin blocks. All tissue samples were stored and transported on ice at all times until freezing or tissue dissociation to minimize any transcriptional degradation.

### Preparation of node samples

For the SAN, a 6 × 3 cm portion of the posterolateral RA with its long axis parallel to and centred on the crista terminalis was dissected. This was then divided into 5-mm-thick strips cut perpendicular to the crista terminalis (Supplementary Fig. 1a). For the AVN region, a tissue sample including the triangle of Koch (bordered by Todaro’s tendon, the coronary sinus ostium and the tricuspid valve annulus) as well as the basal septum (spanning from the interatrial to interventricular septum and including the membranous septum) was dissected (Supplementary Fig. 1b). As before, the sample was then divided into strips that were cut perpendicular to the tricuspid annular plane. Each strip was then embedded in OCT medium and frozen, retaining information of its position in the septum–lateral wall axis. As confirmed by H&E staining, the lateral portions captured AV nodal tissue, whereas the septal portions included the AVB and its branches.

### Single nuclei isolation

Single nuclei were obtained from flash-frozen tissues using sectioning and mechanical homogenization as previously described^2,54^. Slices of 5–10 mm thickness from frozen tissue were first sectioned with a cryostat in a 50-μm thickness section. All sections from each sample were homogenized using a 7 ml glass Dounce tissue grinder set (Merck) with 8–10 strokes of a loose pestle (A) and 8–10 strokes of a tight pestle (B) in homogenization buffer (250 mM sucrose, 25 mM KCl, 5 mM MgCl_2_, 10 mM Tris-HCl, 1 mM dithiothreitol (DTT), 1× protease inhibitor, 0.4 U μl^−1^ RNaseIn, 0.2 U μl^−1^ SUPERaseIn and 0.1% Triton X-100 in nuclease-free water). Homogenate was filtered through a 40 μm cell strainer (Corning). After centrifugation (500*g*, 5 min, 4 °C), the supernatant was removed and the pellet was resuspended in storage buffer (1× PBS, 4% BSA and 0.2 U μl^−1^ Protector RNaseIn). Nuclei were stained with 7-AAD viability staining solution (BioLegend), and positive single nuclei were purified by FACS using a MA900 Multi-Application Cell Sorter (Sony) and its proprietary software (Cell Sorter v.3.1.1) (Supplementary Fig. 7). Nuclei purification and integrity were verified by microscopy, and nuclei were further processed for multiome paired RNA and ATAC-seq using Chromium Controller (10x Genomics) according to the manufacturer’s protocol.

### Chromium 10x library preparation

Single nuclei were manually counted by Trypan blue exclusion. Nuclei suspension was adjusted to 1,000–3,000 nuclei per microlitre and loaded on a Chromium Controller (10x Genomics) with a targeted nuclei recovery of 5,000–10,000 per reaction. Next, 3′ gene expression libraries and ATAC libraries were prepared according to the manufacturer’s instructions from Chromium Single Cell ATAC and multiome ATAC+Gene Expression kits (10x Genomics). Quality control of cDNA and final libraries was done using Bioanalyzer High Sensitivity DNA Analysis (Agilent) or a 4200 TapeStation System (Agilent). Libraries were sequenced using a NovaSeq 6000 (Illumina) at the Wellcome Sanger Institute with a minimum depth of 20,000–30,000 read pairs per nucleus.

### Visium slides and library preparation

For fresh-frozen samples, samples were frozen and embedded in OCT medium using a dry ice-cooled bath of isopentane at −45 °C. OCT-embedded samples were sectioned using a cryostat (Leica CX3050S) and were cut at 10 μm.

For formalin-fixed paraffin-embedded (FFPE) samples, fresh samples were fixed in >5 times their volume of 4% v/v formalin at ambient temperature for 24 h before processing to paraffin on a Tissue-Tek Vacuum Infiltration Processor 5 (Sakura Finetek). FFPE blocks were sectioned at 5 μm using a microtome (Leica RM2125RT).

Samples of the microanatomical regions of interest (ROIs) were selected on the basis of morphology with expert review (S.Y.H.), orientation (based on H&E staining) and either RNA integrity number (fresh-frozen samples) or DV200 (formalin-fixed) that was obtained using an Agilent 2100 Bioanalyzer. In addition, FFPE tissues were checked for possible detachment issues using 10x Genomics Adhesion test slides. FFPE Visium Spatial Gene Expression (10x Genomics) was performed following the manufacturer’s protocol. For fresh-frozen samples, the Tissue Optimization protocol from 10x Genomics was performed to obtain a permeabilization time of 45 min, and the FF Visium Spatial Gene Expression experiment was performed as per the manufacturer’s protocol (10x Genomics). H&E-stained Visium Gene Expression slides were imaged at ×40 magnification on a Hamamatsu NanoZoomer S60. After transcript capture, the Visium Library Preparation protocol from 10x Genomics was performed. Eight cDNA libraries were diluted and pooled to a final concentration of 2.25 nM (200 μl volume) and sequenced on 2× SP flow cells of an Illumina NovaSeq 6000.

### Read mapping

After sequencing, samples were demultiplexed and stored as CRAM files. Each sample of sc/snRNA-seq was mapped to the human reference genome (GRCh38-3.0.0) provided by 10x Genomics and using the CellRanger software (v.3.0.2) or STARsolo (v.2.7.3a) with default parameters. For single nuclei samples, the reference for pre-mRNA was created using the method provided by 10x Genomics (https://support.10xgenomics.com/single-cell-gene-expression/software/pipelines/latest/advanced/references). Each sample of multiome, or Visium, were mapped to the human reference genome (multiome: GRCh38-2020-A-2.0.0; Visium: GRCh38-3.0.0) provided by 10x Genomics using CellRanger ARC (v.2.0.0) or SpaceRanger (v.1.1.0) with default parameters. For Visium samples, SpaceRanger was also used to align paired histology images with mRNA capture spot positions in the Visium slides. Part of the SAN samples were mixed with different donors after the nuclei isolation for cost-efficient experimental design (Supplementary Table 8) and computationally demultiplexed (Soupercell, v.2.0)^55^ on the basis of genetic variation between the donors.

### Quality control and processing of data

For sc/snRNA-seq and multiome gene expression data, the CellBender algorithm^56^ (remove-background) was applied to remove ambient and background RNA from each count matrix produced using the CellRanger pipeline. Python (v.3), Pandas (v.1.3.5), NumPy (v.1.21.5), Matplotlib (v.3.5.2) and Scanpy (v.1.8.2 and v.1.9.1) were used for quality control and downstream processing. Cells or nuclei for each sample were filtered for more than 200 genes and less than 20% (cells) or 5% (nuclei) mitochondrial and ribosomal reads. A Scrublet^57^ (v.0.2.3) score cutoff value of 0.3 of was applied to remove doublets. The Scanpy toolkit was used to perform downstream processing.

For multiome ATAC data (10x Genomics), the data processed using CellRanger ARC were further analysed using ArchR (v.1.0.2)^58^. Quality control was performed, considering, among other factors, transcription start site enrichment, nucleosomal banding patterns, the number and fraction of fragments in peaks, reads falling into ENCODE blacklist regions as well as doublet scores computed by ArchR. For high-quality cells, reads were mapped to 500-bp bins across the reference genome (hg38) (TileMatrix). Gene scores based on chromatin accessibility around genes were computed from TileMatrix using the createArrowFiles function to check their consistency with measured expression values. Before peak calling, pseudo-bulk replicates were generated (addGroupCoverages) for each fine-grained cell state annotated using the paired gene expression data. Peak calling (501 bp fixed-width peaks) was performed for each cell state, and the peak sets were merged to obtain a unified peak set (addReproduciblePeakSet). A cell-by-peak count matrix was obtained using the addPeakMatrix function.

For Visium data, the Scanpy toolkit was used for quality control and downstream processing. Visium spots of each sample were filtered for more than 500 UMI counts and 300 genes.

### Data integration and cell-type annotation

All transcriptome data were integrated using scVI^59^ (v.0.14.5, n_hidden=128, n_latent=50, n_layers=3, dispersion=‘gene-batch’) and scArches^60^ (v.0.5.5, n_hidden=128, n_latent=50, n_layers=3, dispersion=‘gene-batch’) with correcting batch effects (donor, cells or nuclei, and 10x Genomics library generation kits) and removing unwanted source of variations (total counts, per cent mitochondrial genes and per cent ribosomal genes for ‘continuous_covariate_keys’). Scanpy functions were used to compute a neighbourhood graph of observations based on the scVI latent space (scanpy.pp.neighbors) and to perform dimensionality reduction (scanpy.tl.umap) and Leiden clustering (scanpy.tl.leiden, resolution = 1.0). Clusters showing hybrid transcriptional signatures that also had a high scrublet score were removed. After re-clustering, cell lineages were annotated on the basis of the expression of major marker genes and statistically identified marker gene expression for each cluster (scanpy.tl.rank_genes_groups).

To identify fine-grained cell states of the CCS, aCMs of the SAN were subclustered; thus we identified a cluster of P cells (SAN_P_cell) that expressed canonical channel genes (*HCN1* and *HCN4*)^61^ and a TF (*TBX3*) (Fig. 1c and Extended Data Fig. 2a). Subclustering of AVN aCMs and vCMs showed two CCS cell state clusters (AVN pacemaker cell; AVN_P_cell and AVB cell; AV_bundle) (Fig. 1d). AVB cells formed a distinct cluster defined by an enrichment in *GJA5* (which encodes Cx40), *CRNDE* and *CNTN5*, which were previously identified as a marker of His bundle cells in the mouse heart^9^ (Extended Data Fig. 2c). To identify Purkinje cells, CM populations from AVN samples were integrated and clustered with those from the AX. This analysis showed one cluster (cluster 8) that contained not only the CCS cells from the AVN but also a population derived from the AX expressing Purkinje cell marker genes (*GJA5, IRX3, KCNJ3* and *MYL4*)^62^ (Fig. 1e and Extended Data Fig. 2d,e).

Cell states of other cell types and other regions were defined by label transfer (scNym)^63^ using a published dataset^2^ as a reference with revised annotations. The new annotations include neural cell populations, which express pan-glial markers and lack core neuronal markers (Extended Data Fig. 2i); therefore this compartment is best described as glia and will be described below with the ‘_glial’ suffix. FB4 was renamed as FB4_activated based on FB-activation signature genes (*POSTN* and *TNC*) and genes encoding ECM proteins (*COL1A1*, *COL1A2*, *COL3A1* and *FN1*) (Extended Data Fig. 2h,j). vCM3 was renamed as vCM3_stressed based on the specific expression of *NPPB*, which encodes B-type natriuretic peptide (BNP), which is a diagnostic marker for HF and a valuable prognostic predictor for the entire spectrum of disease severity and expressed in stressed CMs^64^ (Extended Data Fig. 2h,k). EC7_atria was renamed as EC7_endocardial based on a recently published study^45^. For myeloid cells, dimensionality reduction and batch correction (scVI) with Leiden clustering were repeated to identify and annotate fine-grained cell states, such as tissue-resident LYVE1^+^ MPs^65^, based on the markers (Supplementary Fig. 2c,d). The transferred cell state labels that were not consistent with the coarse-grained cell-type labels based on the global clusters were replaced with ‘unclassified’ and excluded from downstream analyses.

snATAC-seq data were integrated using cell-by-peak count matrix and peakVI^66^ (v.0.19.0) with correction for batch effects (donor). Scanpy functions were used to compute a neighbourhood graph of observations based on the peakVI latent space (scanpy.pp.neighbors) and to perform dimensionality reduction (scanpy.tl.umap).

### Spatial mapping of cell states with cell2location

To spatially map heart cell states defined by single-cell transcriptomics data analysis in the Visium data, we used our cell2location (v.0.1) method^11,67^. In brief, we first estimated reference signatures of cell states using sc/snRNA-seq data of each region and a negative binomial regression model provided in the cell2ocation package. For the cell types that had fewer than 100 cells or nuclei per region, cells or nuclei from all the regions were used for the reference signature inference. The inferred reference cell state signatures were used for cell2location cell-type mapping for corresponding regions that estimate the abundance of each cell state in each Visium spot by decomposing spot mRNA counts. The H&E images of the Visium slides were used to determine the average number of nuclei per Visium spot (*n* = 7) in the tissue and used as a hyperparameter in the cell2location pipeline. For Visium–FFPE sections (Extended Data Fig. 6c,d), cell state proportions in each Visium spot were calculated based on the estimated cell state abundances.

### Cell state spatial enrichment analysis

Anatomical microstructures of spatial transcriptomics data were manually annotated using the paired histology images as follows: epicardium, subepicardium, endocardium, myocardium, vessel, nerve, adipose tissue, cardiac_skeleton, fibrosis, node, AVB and Purkinje cell. Cell state proportions per spot were calculated based on the estimated abundance of cell states (cell2location). Cell state enrichments (odds ratio) in each structure were calculated by dividing the odds of target cell state proportions by the odds of the other cell state proportions. Odds of cell proportions were calculated as the ratio of cell proportion in the spots of a structure of interest to that in the other spots. Significance was obtained by chi-square analysis (scipy.stats.chi2_contingency) and the *P* value was corrected using the Benjamini–Hochberg method.

The mapping of expected cell types to histologically defined structures such as EC7_endocardial in the endocardium, glial cells (NC1_glial) in the nerve, arterial smooth muscle cells (SMC2_art) in the vessel, and FBs and MPs in fibrosis structures (Supplementary Fig. 3a–d) provided further validation of the spatial mapping method.

### Cellular microenvironment discovery

The NMF analysis implemented in the cell2location pipeline was performed on the spatial mapping results of each anatomical region of the heart. The NMF model was trained for a range of cell state combinations (number of factors: n_fact) *N*={5,...,14}, and the effect size of the cell state group abundance between the spots within a given structure against the spots in the other areas was calculated for each factor (95 factors in total) (Supplementary Fig. 4, step 1). To test the significance, we permuted the annotation labels of all spots and generated a null distribution of the effect size. The *P* values were calculated on the basis of the proportion of the value that is as high as or higher than the actual effect size. For each given structure, we first selected the factor that has the highest significant effect size (best-factor) (Supplementary Fig. 4, step 2). Next, we selected the n_fact that had multiple numbers of factors (fine-factors) with an effect size more than an arbitrary proportion (0.5) of the best-factor (Supplementary Fig. 4, step 3). We considered the fine-factors as refined microenvironments, which were identified using the NMF method (and not with the knowledge-based structural annotations).

For the myocardial-stress niche, first the estimated abundance values (cell2location) of FB4_activated and vCM3_stressed cells were multiplied. Based on the multiplied abundance, clusters of neighbouring spots (*n* > 5) that had higher than a value of (0.03) were selected.

### CellPhoneDB neural–GPCR expansion module

Using the HUGO Gene Nomenclature Committee (HGNC)^68^ library of GPCRs as a master list (HGNC group 139), we used publicly available databases (UniProt, Reactome, IUPHAR and GPCRdb (https://gpcrdb.org/)^69^) to generate a set of GPCRs with known ligands. To generate ligand–receptor interactions for these GPCRs, we used ligand genes (for gene-encoded ligands). For non-gene-encoded ligands (such as small-molecule ligands), we used ligand proxies in the form of specific biosynthetic enzymes or transporter genes. Additionally, we added new trans-synaptic adhesion molecule interactions^29,70,71^. Together, this formed more than 800 new interactions (Supplementary Tables 3 and 4), which we used with the user-defined ‘database generate’ function in CellPhoneDB^7^.

### Spatially resolved cell–cell interaction analysis

CellPhoneDB^7,72^ analyses with a custom neural–GPCR expansion module were performed on the identified niches and the cell state components. Overall, the cell–cell interaction inferences were performed using single-cell transcriptomics data of each anatomical region and by restricting to the cell states that were colocalized in the identified cellular niches.

For CCS and myocardial-stress niches, we selected the cell states that were either in the node of the SAN or the AVN and retrieved the interacting pairs of ligands and receptors that satisfied the following criteria: (1) all the members were expressed in at least 10% of the cells in the cell states; and (2) ligand–receptor complexes specific to two cell states were inferred by the statistical method framework in CellPhoneDB (‘statistical_analysis’, *P* value threshold = 0.05).

For epicardial–subepicardial niches, the ligand–receptor interactions of the colocalized cell states were retrieved on the basis of the following criteria: (1) all the members were expressed in at least 10% of the cell states; and (2) at least one of the members in the ligand or the receptor was a differentially expressed gene (DEG) compared with other cell states (scanpy.tl.rank_genes_groups, *P* value threshold = 0.05, log_2_(fold change) threshold = 0.1). The ligand–receptor interactions were further selected on the basis of mean expression levels and the biological questions as indicated in the Results and the figure legends.

The following HGNC annotations were used for selecting some of the ligand–receptor interactions: chemokines (HGNC GID:189), cytokines (HGNC GID:599, 602, 1932, 781 and 1264), and LGIC (HGNC GID:GID161).

The following cell states were used in each analysis: SAN (SAN_P_cell, FB2, FB4_activated, FB5, FB6, NC2_glial_NGF^+^ and LYVE1^+^IGF1^+^ MP); AVN (AVN_P_cell, aCM2, FB1, FB2, FB5, SMC1_basic, SMC2_art, NC2_glial_NGF^+^, LYVE1^+^IGF1^+^ MP and mast); epicardial–subepicardial niche (meso, EC6_ven, EC8_ln, PC2_atria, LYVE1^+^IGF1^+^ MP, B_plasma, T/NK_cycling, FB2, FB3, FB5 and NC1_glial); and myocardial-stress niche (FB3, FB4_activated, FB5, vCM3_stressed, EC2_cap, EC3_cap, EC4_immune, EC6_ven, PC3_str, SMC2_art, LYVE1^+^IGF1^+^ MP, MoMP and NK_CD16hi).

### Ion channel and GPCR profile

Differential gene expression analysis with *t*-test method was performed using the Scanpy function scanpy.tl.rank_genes_groups. Only multiome gene expression data were used to avoid the technical batch effects due to kit differences. *P* value correction was performed using the Benjamini–Hochberg method. Each of the CCS cells (SAN_P_cell, AVN_P_cell, AV_bundle_cell and Purkinje cells) was compared with non-CCS aCMs as a reference (Supplementary Table 2). Genes were deemed differentially expressed with an adjusted *P* value of < 0.05. DEGs encoding for ion channels and GPCRs were selected based on HGNC groups 177 and 139, respectively. Upregulated (log_2_(fold change) > 0) DEGs were depicted in the GPCR overview schematic (Fig. 3b). To compare the transcriptional similarity of working (aCM, vCM) and CCS cell states, a dendrogram based on principal-component-analysis-reduced gene expression was computed using the Scanpy function scanpy.tl.dendrogram (Extended Data Fig. 8c).

### Mouse DEG analysis

A list of upregulated ion channel genes (log_2_(fold change) > 1, adjusted *P* value < 0.01) for SAN_P_cells was made. Differentially expressed testing summary statistics from two mouse single-cell studies were obtained^52,73^. In both studies, differentially expressed testing was conducted by comparing sinoatrial CMs (P cells) against all other cells. Genes orthologous to the list of upregulated (human) P cell genes were identified in the mouse differentially expressed summary statistics (using the NCBI Orthologs database as a reference). The differentially expressed statistics (log_2_(fold change), –log(*P* value)) from the mouse are plotted. Human and mouse data were not integrated.

### Identification of ligands in Visium data

Four spatial transcriptomics, sections were identified as containing the RAGP by an expert anatomist (S.Y.H.). Spot counts from the Visium–FFPE sections that contained RAGP were normalized, and spots were scored for the expression of four generic pan-neuronal cytoskeletal markers (*PRPH*, *NEFL*, *NEFM* and *NEFH*) using the SCANPY sc.tl.gene_score() function. Correlation of individual gene expression profiles with this score was calculated (Pearson *r* and *P* value for each gene). The ligand/ligand-proxy list created as part of the CellPhoneDB neural–GPCR module was used to identify ‘ligand’ genes among the set of correlated genes.

### Gene regulatory network

The Scenic pipeline^74,75^ was used (pySCENIC, v.0.11.2) to predict TFs and putative target genes regulated in P cells. First, gene regulatory interactions were calculated based on co-expression (either positively or negatively correlated) across the single-cell transcriptomics datasets of aCMs (using only multiome data) with GRNBoost2 (ref. ^76^). This was followed by pruning interactions using known TF-binding motifs and the construction of dataset-specific regulatory modules (regulons)^77^. Regulons were then scored in each individual cell using AUCell. P-cell-relevant TFs and target genes were retrieved based on the following criteria: (1) regulator TFs that are DEGs in P cells compared with other aCMs (scanpy.tl.rank_genes_groups, with only multiome data, *P* value threshold = 0.05, log_2_(fold change) threshold = 0.5); (2a) for activating regulons, target genes that were expressed in at least 10% and differentially expressed in P cells compared with other aCMs (same criteria as TF selection); and (2b) for repressor regulons, target genes that were expressed at low levels in P cells compared with other aCMs (*P* value threshold = 0.05, log_2_(fold change) threshold = −0.5). A network of regulatory TFs and target genes was then constructed by linking individual regulons to create a graph (NetworkX, v.2.6.3) (Fig. 3c and Extended Data Fig. 8i). The node colour of the target genes is based on the class GPCR (HGNC and GID139), ion channel (HGNC and GID177) or TFs^78^.

The interactions of regulatory TFs and target genes were also inferred using the snATAC-seq data and ArchR (v.1.0.2)^58^. TF-binding motifs in the identified peaks were searched (addMotifAnnotations, motifSet=“cisbp”), and correlations between peak accessibility and gene expression were analysed (addPeak2GeneLinks and getPeak2GeneLinks, correlation > 0.2 or < −0.2, FDR < 1 × 10^–4^) using the multiome data of aCMs. TFs and potential target gene interactions were obtained by combining the two results (Supplementary Fig. 5c). The inferred interactions are highlighted in red in the activator network graph (Fig. 3c) and blue in the repressor network graph (Extended Data Fig. 8i).

### GWAS SNP enrichment analysis

To create a list of SNPs associated with various physiological and pathological cardiovascular traits, index SNPs (meeting genome-wide significance) were first extracted from the NHGRI-EBI GWAS Catalog^79^ using the R package gwasrapidd^80^. Index SNPs were added to by SNPs in tight linkage disequilibrium (*r*^2 ^> 0.8) based on 1000 Genomes (phase 3) European samples, obtained using the Ensembl API^80,81^, with the window size set to the default 500 kb.

For each nucleus barcode in the snATAC-seq dataset, counts of each identified peak was binarized (1 if the read count was >0). The binarized barcode by peak matrix was then aggregated by cell state (defined using the paired gene expression data) to form a cell state-by-peak matrix, in which a peak was defined as open for a given cell state if the binarized count was 1 in at least 5% of that population.

To calculate enrichment, a permutation test was performed using a previously described method^82^. For each cell state, a random background was created by shuffling the open/closed labels of the peaks of that cell state such that a random set of peaks were annotated as open, the number of which equalled the actual number of open peaks for that cell state. This was repeated to create 1,000 random permutations. For each trait and cell state, the proportion of trait-associated SNPs falling within the open peaks of that cell state was calculated (the SNP proportion). This SNP proportion was also calculated for each of the 1,000 random permutations. A *P* value could then be calculated as the fraction of times the random SNP proportion exceeded or was equal to the real SNP proportion. Finally, these *P* values were corrected for multiple testing using the Benjamini–Hochberg method.

### Drug2cell

Drug and target gene information was obtained from ChEMBL^83,84^ (v.30). Drugs were filtered based on targeting organisms (*Homo sapiens*), achieved phase in a clinical trial (max_phase=4, clinically approved), and functionally active or not. The activity (pChEMBL) threshold was specifically set for each family of target molecules according to the IDG project (https://druggablegenome.net/ProteinFam) (kinases: ≦30 nM; GPCRs: ≦100 nM; nuclear hormone receptors: ≦100 nM; ion channels: ≦10 μM; others: ≦1 μM) (Supplementary Table 9). Clinically approved drugs were categorized based on the WHO ATC classification (https://www.who.int/tools/atc-ddd-toolkit/atc-classification). Drug scores in each single cell were calculated based on the target gene expression levels. Score were obtained by taking the mean of a set of target genes either without (method=’mean’) or with (method=’seurat’) subtracting with the mean expression of a reference set of genes^85^. The reference set was randomly sampled from the gene pool for each binned expression value. For the drug repurposing analysis, all the drugs tested and selected were ones that had the statistically highest score in a cell type of interest by testing with Wilcoxon sum test, and *P* values were adjusted using the Benjamini–Hochberg method. For the drugs targeting GPCRs or ion channels, we searched the clinically approved (maximum phase: 4) and preclinical bioactive molecules with drug-like properties (maximum phase: 1–3) that target genes encoding GPCRs (HGNC GID:139) or ion channels (HGNC GID:177). The drug2cell Python package is available at GitHub (https://github.com/Teichlab/drug2cell).

### In vitro validation of chronotropic effects of GLP-1

#### Cell culture

hiPSC-derived CMs (hiPSC-CM) were differentiated and maintained as previously described^86,87^. In brief, IMR90 hiPSCs from WiCell^88^ were seeded onto plates coated with Matrigel (Corning, 356231) in TeSR-E8 medium (StemCell Technologies, 05990) with 10 μM Y-27632 dihydrochloride (Sigma-Aldrich, Y0503). The next day, the medium was switched to TeSR-E8 without Y-27632. Media were subsequently changed daily. Before starting the differentiation into CMs, the cells were replated twice using 0.5 mM EDTA (Thermo Fisher, 15575020, diluted 1:1,000 in PBS (Gibco, 20012027)) at room temperature for 6 min to detach them before plating. For the differentiation process, cells that reached 90% confluency were treated for 2 days with 5 μM CHIR-99021 (Tocris, 4423) in RPMI 1640 (Gibco, 11875119) supplemented with B27, minus insulin (Gibco, A1895602). On day 2, the cell culture medium was replaced with RPMI/ B27, minus insulin. For the next 2 days, 2 μM Wnt-C59 (Biorbyt, orb181132) was added in RPMI/B27, minus insulin. Cells were then cultured in RPMI/B27, minus insulin, with media changes every 2 days. hiPSC-CM contraction was observed between days 8 and 10. On day 11, the cells were placed in starvation medium (RPMI without glucose (Gibco, 11879020) supplemented with B27 (Gibco, 17504044)) for 2 days to improve purity. On day 15, the cells were detached using TrypLE select enzyme (10×) (Life Technologies, A1217702) and replated at a density of 2 × 10^6^ per well in RPMI/B27, 10% KOSR (Thermo Fisher, 10828028) and 10 μM Y-27632 dihydrochloride. The cells were then cultured in RPMI/B27, and media were changed every 2 days.

#### Gene expression analysis of hiPSC-CMs

Bulk RNA-seq data from IMR90-derived CMs, deposited into the Sequence Read Archive public repository with accession number PRJNA629893, were analysed as previously described^89^. Transcripts per million (TPM), indicating the normalized amplitude of gene expression, were used to examine gene expression. The TPM was calculated by dividing the read counts by gene length and the total number of exon reads, and then multiplied by the scaling factor of 1,000,000 (ref. ^90^).

#### Calcium imaging and quantification

hiPSC-CMs were stained for calcium imaging 30–35 days after the differentiation protocol was started. Cells were seeded in 96-well plates at 100,000 cells cm^–2^ density 1 week before imaging and staining. On the day of imaging, cells were gently washed with phenol-red-free RPMI (Thermo Fisher, 11835063), then stained with 1 μM Fluo-4 (Thermo Fisher, F14201) solution and incubated at 37 °C for 40 min. After incubation, the Fluo-4 solution was replaced with fresh, pre-warmed phenol-red-free RPMI. Then, hiPSC-CMs were transferred to a microscopy humidified chamber (pre-set at 37 °C with 5% CO_2_) and allowed to acclimatize for 10 min. Cells were imaged using a Zeiss Axio Observer inverted widefield microscope with a ×20/0.8 objective. The time series experiment was performed with 10 ms exposure time on the Fluo-4 channel (excitation 494 nm, emission 516 nm), and recorded for 10 s at 100 f.p.s. Stage positions for each well were stored to allow recurrence after drug treatment. All wells were scanned for baseline calcium transients. Then, hiPSC-CMs were treated with ivabradine (Sigma, SML0281), GLP-1 (Tocris, 2082) or the corresponding vehicles (DMSO for ivabradine and water for GLP-1). After 20 min, cells were scanned again using the same configuration.

Quantification of calcium imaging videos was performed using Fiji (v.2.1.0). To correct the intensity decay due to photobleaching, the Fiji bleach correction plugin (exponential fit method) was applied to the raw image series. Then, corrected image series were divided by their minimum intensity to remove background. A confluent region (containing at least 20 cells) was selected for quantification. Spiky plugin (https://github.com/PCCV/Spiky)^91^ was used to automate peak detection and quantification. Amplitude (peak value minus baseline value), Pk2Pk (time between two consecutive peaks), Time2Pk (time between threshold to the peak) and RW_50_ (time at 50% of amplitude from peak to next baseline at right) were averaged from multiple peaks detected. All results were normalized to the corresponding baseline values.

Statistical tests were performed in Prism 9 using D’Agostino and Pearson test to test for normal distribution. Unpaired *t*-test was used to compare vehicle and treated groups. *P* < 0.05 was regarded as significant.

#### Immunofluorescence staining of cells

hiPSC-CMs were fixed in 3.7% formalin in PBS then either blocked and permeabilized in 4% (w/v) BSA (Sigma Aldrich, A3059) and 0.2% (v/v) Triton X-100 (Thermo Fisher, 85111) in PBS (Gibco, 20012027) (for HCN1 and HCN4 staining) or blocked in 1% (w/v) BSA and 5% (v/v) normal goat serum (EDM Millipore, S26-100ML) in PBS (for GLP1R staining) for 30 min at room temperature. Incubation with primary antibodies (Supplementary Table 10) diluted in BSA, Triton X-100 and PBS (HCN1 and HCN4) or BSA, goat serum and PBS (GLP1R) was done overnight at 4 °C. Isotype controls and secondary antibody only (Supplementary Table 10) stainings were performed as negative controls. Following three washes with PBS, cells were stained with secondary antibodies diluted in BSA, Triton X-100 and PBS or BSA, goat serum and PBS was done for 1 h at room temperature (Supplementary Table 10). After three washes in PBS, cell nuclei were stained with DAPI (Invitrogen, D1306) for 15 min at room temperature. DAPI was rinsed and PBS was added to the cells. Confocal imaging acquisition was performed using a Zeiss LSM-780 inverted microscope with a EC Plan Neofluar ×40/1.3 oil objective at the Imperial College London Hammersmith FILM facility using 405 nm, 488 nm and 633 nm lasers for excitation. Image processing was performed in Fiji (v.2.1.0).

### smFISH

The FFPE-embedded heart tissue sections (with a thickness of 5 μm) were placed onto SuperFrost Plus slides. Staining with a RNAscope Multiplex Fluorescent Reagent kit v2 assay (Advanced Cell Diagnostics, Bio-Techne) was automated using a Leica BOND RX, according to the manufacturer’s instructions. The tissues were baked and dewaxed on the Leica Bond RX, followed by the application of a heat-induced epitope retrieval step with epitope retrieval 2 for 15 min at 95 °C and protease digestion with protease III for 15 min. Subsequent processing included RNAscope probe hybridization and channel development with Opal 520, Opal 570 and Opal 650 dyes (Akoya Biosciences) at a concentration of 1:1,000, and streptavidin-conjugated Atto-425 (Bio Trend) at a concentration of 1:400 using TSA-biotin (TSA Plus Biotin Kit, Perkin Elmer). All nuclei were DAPI stained. Stained sections were imaged using a Perkin Elmer Opera Phenix High-Content Screening System with a ×20 water-immersion objective (NA of 0.16, 0.299 μm per pixel). The following channels were used: DAPI (excitation, 375 nm; emission, 435–480 nm); Opal 520 (excitation, 488 nm; emission, 500–550 nm); Opal 570 (excitation, 561 nm; emission, 570–630 nm); Opal 650 (excitation, 640 nm; emission, 650–760 nm); and Atto 425 (excitation, 425 nm; emission, 463–501 nm). Stained sections were also imaged on a Hamamatsu S60 with a ×40 objective (0.23 μm per pixel).

### RNAscope quantification

Quantification of RNAscope images was performed using ImageJ. To remove background, each channel was initially normalized using the following steps: (1) subtracting the raw image with a Gaussian blur transformation with *σ* = 50; (2) performing a background subtraction with rolling ball radius of 50 pixels; and (3) setting every pixel with an intensity lower than 40 (of an 8-bit image) to 0. Following normalization, each section was quantified with sequential ROIs of 200 × 200 μm. To avoid any bias due to the placement of the initial ROI, quantification was performed over three rounds, with the initial ROI displaced by 50 μm in the *x* and *y* axis in each round. The following parameters were recorded per ROI: number of nuclei; *COL1A1* area; and *NPPB* area.

For data analysis, to avoid variation due to cell density, for each ROI, *COL1A1* and *NPPB* areas were normalized by dividing the area value by the number of nuclei in the ROI. Only ROIs with more than ten nuclei were considered for analysis. An ROI was only considered to contain a *COL1A1* or *NPPB* niche if the staining area was equal to or higher than 1 μm^2^. To avoid any confounding effect due to different section sizes, we quantified the increase in the number of *COL1A1* and *NPPB* niches through the average ROI percentage, consisting of the number of *COL1A1* or *NPPB* niche ROI divided by the total number of ROI, averaged over the three rounds of quantitation. To quantify the expression of *COL1A1* and *NPPB*, we averaged the normalized area of each ROI per niche over the three rounds of quantitation.

### Immunofluorescence staining of tissues

The FFPE-embedded heart tissue samples were sectioned at 6 µm thickness and placed on VWR Superfrost Plus Microscope slides. Deparaffinization was performed in xylene (twice for 10 min), followed by graded washes in 100% ethanol (twice for 10 min), 95% ethanol for 5 min, 70% ethanol for 5 min, 50% ethanol for 5 min, and incubated in deionized water for rehydration. Antigen retrieval was performed using a proteinase K kit (Abcam ab64220) for 5 min at room temperature. Following antigen retrieval, sections were permeabilized and blocked in 0.1 M Tris containing 0.1% Triton X-100 (Sigma), 1% normal mouse serum, 1% normal goat serum and 1% BSA (R&D Systems). Samples were stained for 2 h at room temperature in a wet chamber with the appropriate antibodies, washed three times in PBS and mounted in Fluoromount-G (Southern Biotech). Images were acquired using a TCS SP8 (Leica) inverted confocal microscope with a ×40/1.1 NA water objective. Raw imaging data were processed using Imaris (Bitplane). The antibody information is provided in Supplementary Table 10.

For node and glial cell staining of fresh-frozen heart tissue, 10 µm sections were cut on to Superfrost plus slides and stored at −80 °C. These were thawed for 10 min at room temperature, briefly rehydrated in TBS and fixed with room temperature 4% paraformaldehyde for 5 min. Slides were then immersed for 10 min in TBS, and a hydrophobic pen was used to delimit the area of staining before starting the permeabilization process (0.25% saponin in TBS for 10 min). Blocking buffer (0.3 M glycine in antibody dilution buffer) was applied for 1 h at room temperature, followed by an overnight incubation with primary antibody at 4 °C in a humidified chamber. The primary antibodies used were mouse anti-HCN1 (Abcam, ab84816) at 1:100 dilution and rabbit anti-PLP1 (Abcam ab254363) at 1:500 in 10% normal goat serum in 0.2% Tween-20 and TBS. The isotype controls were Abcam ab37355 and ab172730. Slides were washed three times (5 min each) in 0.2% Tween-20 and TBS with gentle shaking, then secondary antibody solution was applied for 1 h at room temperature in the dark (1:1,000 goat anti-rabbit IgG AF555, LifeTech A21428 and 1:1,000 goat anti-mouse IgG AF647Plus, Fisher 15627898). Slides were washed three times (5 min each) in 0.2% Tween-20 and TBS, incubated with DAPI for 15 min at room temperature in the dark (Invitrogen D1306, 5 mg ml^–1^ stock then diluted 1:50,000), washed again briefly and mounted with ProLong gold antifade mountant (Thermo Fisher). Slides were scanned at ×40 magnification on a Hamamatsu NanoZoomer S60, and ROIs were imaged on a Leica SP8 confocal at ×20 magnification. The antibody information is provided in Supplementary Table 10.

### Reporting summary

Further information on research design is available in the Nature Portfolio Reporting Summary linked to this article.

## Online content

Any methods, additional references, Nature Portfolio reporting summaries, source data, extended data, supplementary information, acknowledgements, peer review information; details of author contributions and competing interests; and statements of data and code availability are available at 10.1038/s41586-023-06311-1.

## Supplementary information

Supplementary Figures This file contains Supplementary Figs. 1–7.

Reporting Summary

Supplementary Table 1 Anonymized donor information, including clinical metadata.

Supplementary Table 2 DE test result of CCS cells. Result of differential gene expression analysis of CCS cells (SAN_P_cell, AVN_P_cell, AV_bundle_cell and Purkinje_cell) compared with the non-CCS aCM (two-sided *t*-test). *P* values were adjusted for multiple comparisons using the Benjamini–Hochberg method.

Supplementary Table 3 CellPhoneDB neural–GPCR module, interactions input. Table of CellPhoneDB neural–GPCR module interactions. Interaction partners use UniProt ID (with protein name also listed) unless they are complexes.

Supplementary Table 4 CellPhoneDB neural–GPCR module, complex input. Table of complexes of CellPhoneDB neural–GPCR module interactions. Complex names are listed, with complex constituents in the same row (using UniProt IDs as identifiers).

Supplementary Table 5 Interactions of activator TF and target genes in P cells. Table of TF and predicted targeted gene pairs that are both highly expressed in P cells compared with other aCMs. log_2_(fold change) of the gene expressions between P cells and other aCMs are shown in the columns “TF_logFC” and “TargetGene_logFC”, respectively.

Supplementary Table 6 Interactions of repressor TF and target genes in P cells. Table of TF and predicted targeted gene pairs that are highly expressed (TF) and lowly expressed (target gene) in P cells compared with other aCMs. log_2_(fold change) of the gene expressions between P cells and other aCMs are shown in the columns “TF_logFC” and “TargetGene_logFC”, respectively.

Supplementary Table 7 Visium gene expression correlation with ‘pan-neuronal cytoskeleton score’. Correlation of genes with the pan-neuronal cytoskeleton score in Visium data (Methods). Pearson’s *r* correlation coefficient (Pearson’s *r*), *P* values and mean expression across all Visium spots. *P* values were corrected for multiple testing using the Benjamini–Hochberg method.

Supplementary Table 8 Information on the samples used in the analyses.

Supplementary Table 9 Filtered drugs and targets. Drugs and target genes from ChEMBL (v.30) were filtered as described in the Methods.

Supplementary Table 10 Antibody information.

## Data Availability

Open-access datasets are available from ArrayExpress (www.ebi.ac.uk/arrayexpress) with accession numbers E-MTAB-12916 (multiome snRNA-seq), E-MTAB-12919 (multiome snATAC-seq) and E-MTAB-12975 (Visium). Processed data of sc/snRNA-seq and Visium data are available for browsing gene expression and download from the Heart Cell Atlas (https://www.heartcellatlas.org). A CellTypist^92^ model trained on this atlas is available for download from the Heart Cell Atlas (https://www.heartcellatlas.org) for automated cell-type annotation of other cardiac sc/snRNA-seq datasets. The CellPhoneDB neural–GPCR expansion module is available from Supplementary Tables 3 and 4 or at GitHub (https://github.com/ventolab/CellphoneDB; CellPhoneDB-database, v.4.1)^93^. The external adult heart sc/snRNA-seq dataset is available from the Human Cell Atlas Data Coordination Platform with accession number ERP123138. The human reference genome (GRCh38) used for read mapping is available from 10x Genomics (https://support.10xgenomics.com/single-cell-gene-expression/software/release-notes/build). The ChEMBL database used for drug2cell analysis is available at the following ftp site: https://ftp.ebi.ac.uk/pub/databases/chembl/ChEMBLdb/releases/chembl_30.

## Code availability

The drug2cell Python package is available at GitHub (https://github.com/Teichlab/drug2cell). The custom code for the other analyses performed in this study is available at GitHub (https://github.com/Teichlab/HCA_Heart_ver2).
