# Author Correction: Spatially resolved multiomics of human cardiac niches

**DOI:** 10.1038/s41586-025-08886-3

**Published:** 2025-03-20

**Authors:** Kazumasa Kanemaru, James Cranley, Daniele Muraro, Antonio M. A. Miranda, Siew Yen Ho, Anna Wilbrey-Clark, Jan Patrick Pett, Krzysztof Polanski, Laura Richardson, Monika Litvinukova, Natsuhiko Kumasaka, Yue Qin, Zuzanna Jablonska, Claudia I. Semprich, Lukas Mach, Monika Dabrowska, Nathan Richoz, Liam Bolt, Lira Mamanova, Rakeshlal Kapuge, Sam N. Barnett, Shani Perera, Carlos Talavera-López, Ilaria Mulas, Krishnaa T. Mahbubani, Liz Tuck, Lu Wang, Margaret M. Huang, Martin Prete, Sophie Pritchard, John Dark, Kourosh Saeb-Parsy, Minal Patel, Menna R. Clatworthy, Norbert Hübner, Rasheda A. Chowdhury, Michela Noseda, Sarah A. Teichmann

**Affiliations:** 1 Wellcome Sanger Institute, Wellcome Genome Campus, Hinxton, Cambridge, UK; 2 National Heart and Lung Institute, Imperial College London, London, UK; 3 Cardiac Morphology Unit, Royal Brompton Hospital and Imperial College London, London, UK; 4 Max Delbrück Center for Molecular Medicine in the Helmholtz Association (MDC), Berlin, Germany; 5 Royal Brompton Hospital, London, UK; 6 Molecular Immunity Unit, Department of Medicine, University of Cambridge, MRC Laboratory of Molecular Biology, Cambridge, UK; 7 Würzburg Institute for Systems Immunology, Max Planck Research Group, Julius-Maximilian-Universität, Würzburg, Germany; 8 Department of Surgery, University of Cambridge, and Cambridge Biorepository for Translational Medicine, NIHR Cambridge Biomedical Centre, Cambridge, UK; 9 Translational and Clinical Research Institute, Newcastle University, Newcastle upon Tyne, UK; 10 Charité-Universitätsmedizin, Berlin, Germany; 11 German Centre for Cardiovascular Research (DZHK), Partner Site Berlin, Berlin, Germany; 12 Department of Physics, Cavendish Laboratory, University of Cambridge, Cambridge, UK

**Keywords:** Computational biology and bioinformatics, Next-generation sequencing, Transcriptomics, Cells

Correction to: *Nature* 10.1038/s41586-023-06311-1 Published online 12 July 2023

In the drug2cell analysis of the initially published article, there was an error in filtering drug and target interactions. The activity (pChEMBL) threshold for these interactions was specifically set for each family of target molecules in accordance with the Illuminating the Druggable Genome (IDG) project. In the original manuscript, there was a discrepancy with the values from the IDG project^1^. We have now updated the threshold values (“Kinases”: revised from ≤1 μM to ≤30 nM; “GPCRs”: unchanged; “Nuclear hormone receptors”: unchanged; “Ion channels”: unchanged; “others”: revised from ≤30 nM to ≤1 μM) in both the analysis reported here and the drug2cell package (https://github.com/Teichlab/drug2cell, version 0.1.2). The main Fig. 4b, Supplementary Table 9 and the relevant section in Methods (“Drug2cell”) have been revised accordingly. In Fig. 4b, two drugs, irbesartan and zopiclone, have been removed from the original figure due to changes in their target molecules. The main text is unaffected and therefore unchanged.
