# Supplementary material for: Spatially resolved multiomics of human cardiac niches
Source: Nature. 2023 Jul 12;619(7971):801–10. doi: 10.1038/s41586-023-06311-1 (PMC10371870; doi:10.1038/s41586-023-06311-1)
Supplement: Supplementary file 1 — This file contains Supplementary Figs. 1–7. [file 41586_2023_6311_MOESM1_ESM.pdf]

nature portfolio

https://doi.org/10.1038/s41586-023-06311-1

Supplementary information

Spatially resolved multiomics of human cardiac niches

In the format provided by the authors and unedited

Lorem ipsum

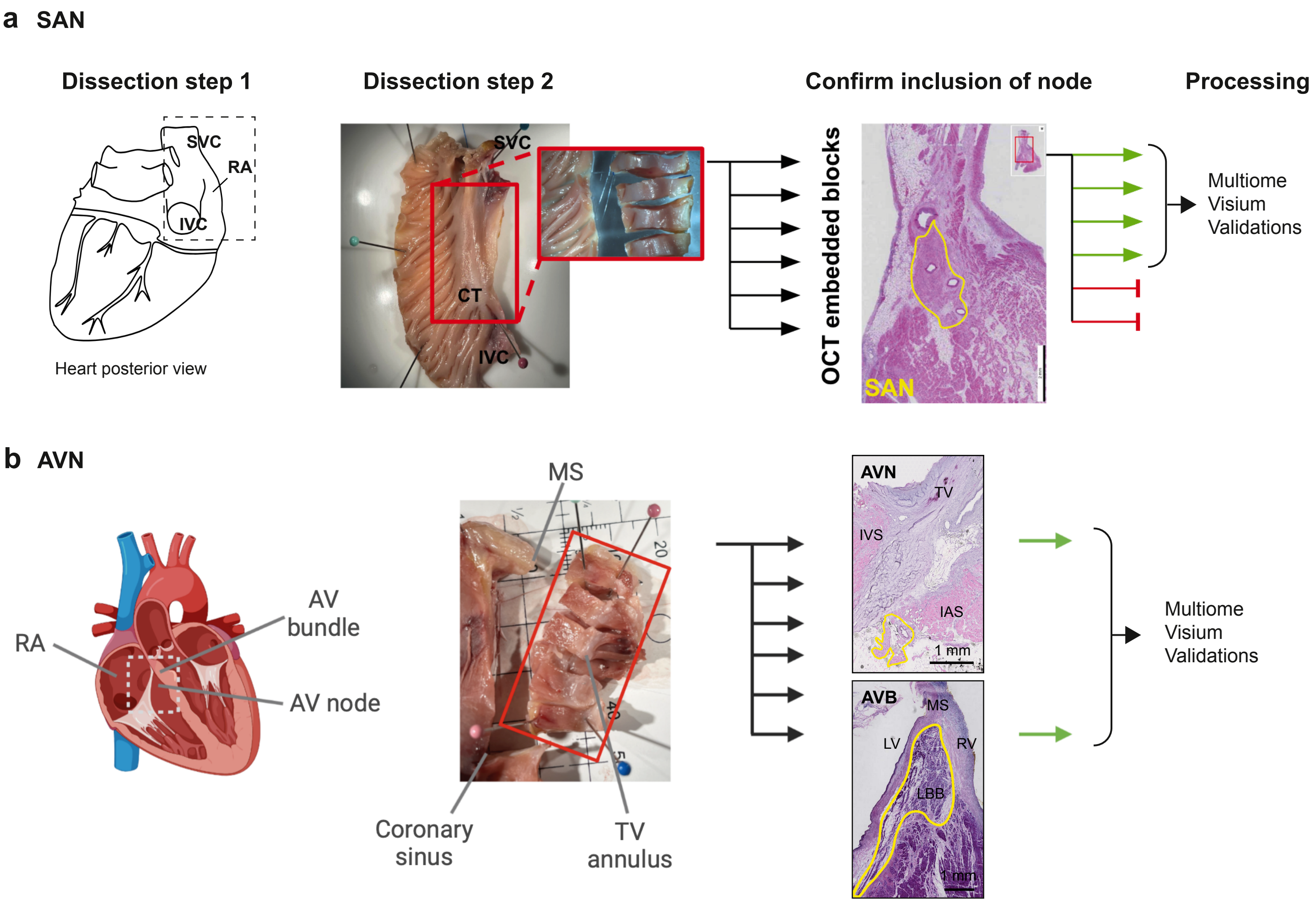

Supplementary Fig. 1 Dissection protocols for cardiac conduction system.
Workflow to capture SAN and AVN regions using a two-stage dissection. For the SAN, a region of the posterolateral RA parallel to and spanning the *crista terminalis* (CT) was isolated (dashed box) (a). For the AVN and AV bundle, a single region incorporating the Triangle of Koch (bordered by Todaro's tendon, the coronary sinus ostium and tricuspid valve annulus) as well as the basal septum (spanning from interatrial to interventricular septum, and including the membranous septum) was isolated (dashed box) (b). These were then subdivided into several smaller samples each ~4mm thick, which were individually frozen and embedded in OCT. Cryosections were stained (HE) with expert histologist review to determine the presence of SAN, AVN and AV bundle (yellow bordered zone). CCS-containing blocks (green arrows) then proceed to Multiome, Visium and RNAscope. SVC (superior vena cava); IVC (inferior vena cava); MS (membranous septum); TV (tricuspid valve). The Illustration in **b** was created using BioRender (https://biorender.com).

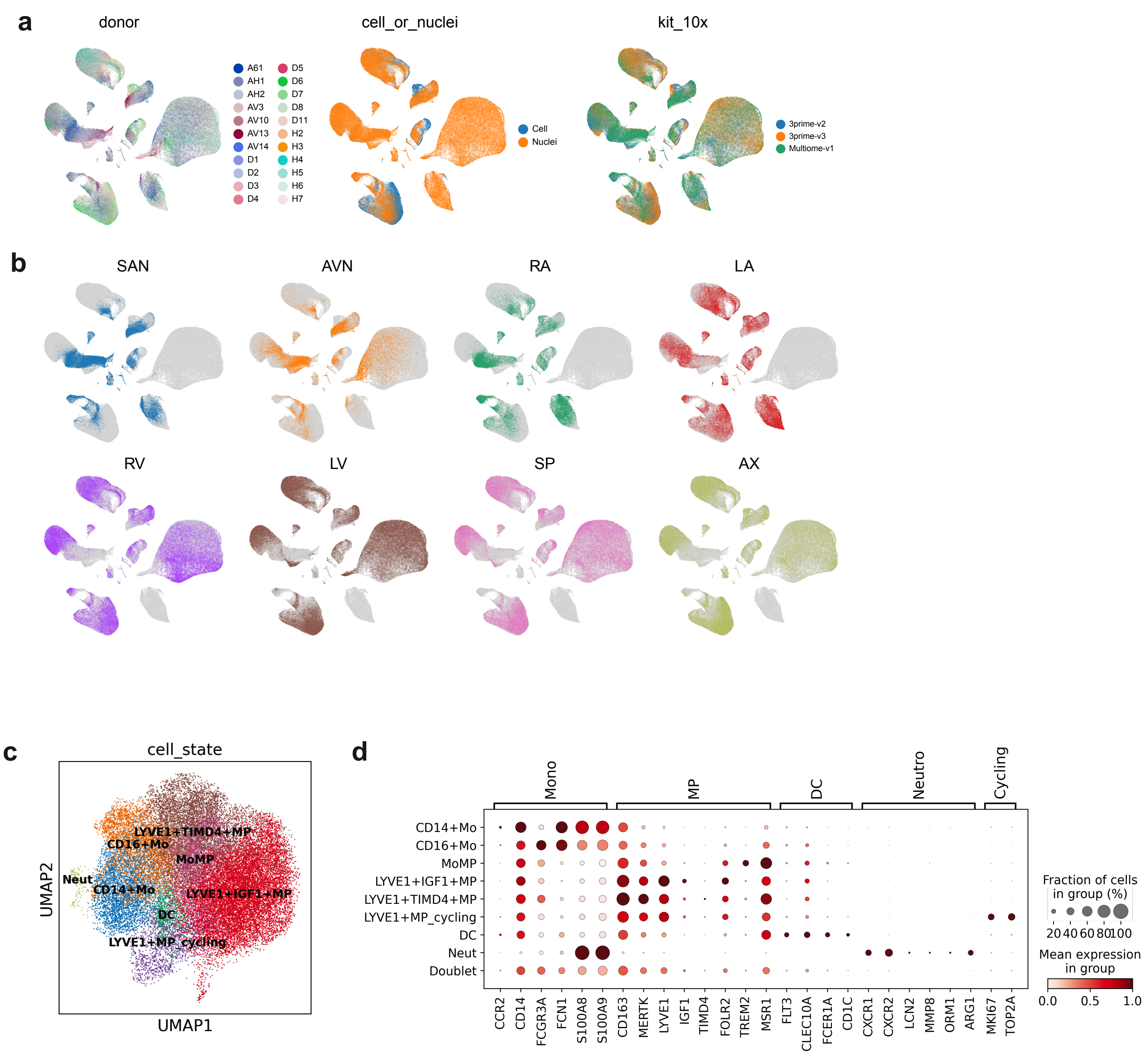

Supplementary Fig. 2 Profiling of cardiac cells.
a,b. UMAP representation of gene expression data from the eight regions showing major batch keys: 'donor', 'cell_or_nuclei', and 'kit_10x' labels (a) or highlighting each region (b).
c,d. Fine-grained cell state annotation of the myeloid compartment. UMAP representation of gene expression of myeloid cells from the eight regions (c). Cell states were annotated based on the clusters and myeloid marker expression (d).

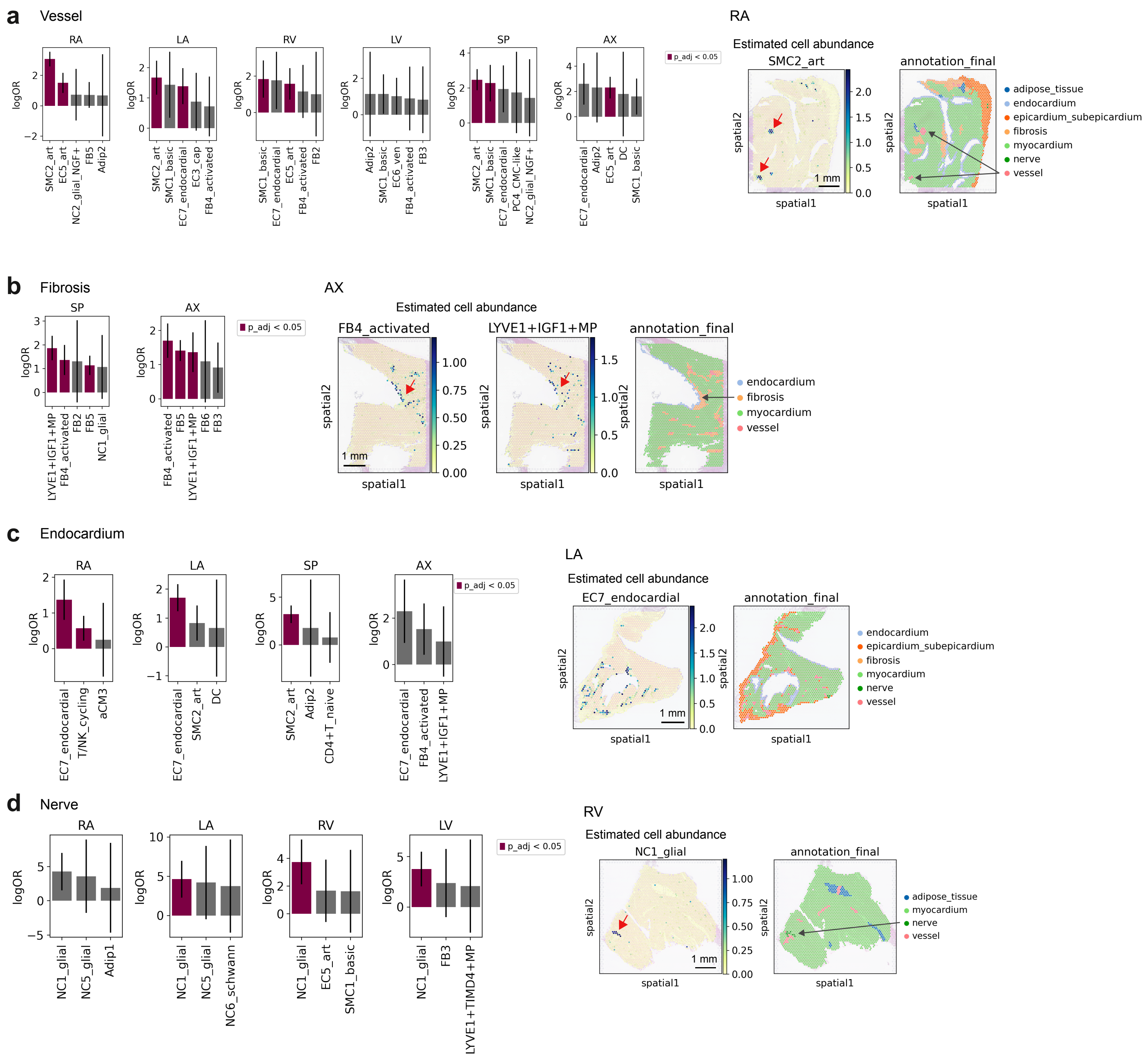

Supplementary Fig. 3 Cell state enrichment in the manually annotated structures.

Cell state enrichments (odds ratio) in the 'vessel' (a), 'fibrosis' (b), 'endocardium' (c), and 'nerve' (d) of the free wall of the six regions: RA, LA, RV, LV, SP, and AX. Statistically significant enrichments (chi-square test, $p<0.05$, p-values were adjusted for multiple comparisons using the Benjamini-Hochberg method) are shown in magenta-pink. Data show log odds ratio with upper and lower 95% confidence interval. Visium spot numbers are RA: 7,027, LA: 5,822, RV: 5,039, LV: 9,626, SP: 8,643, and AX: 6,497.

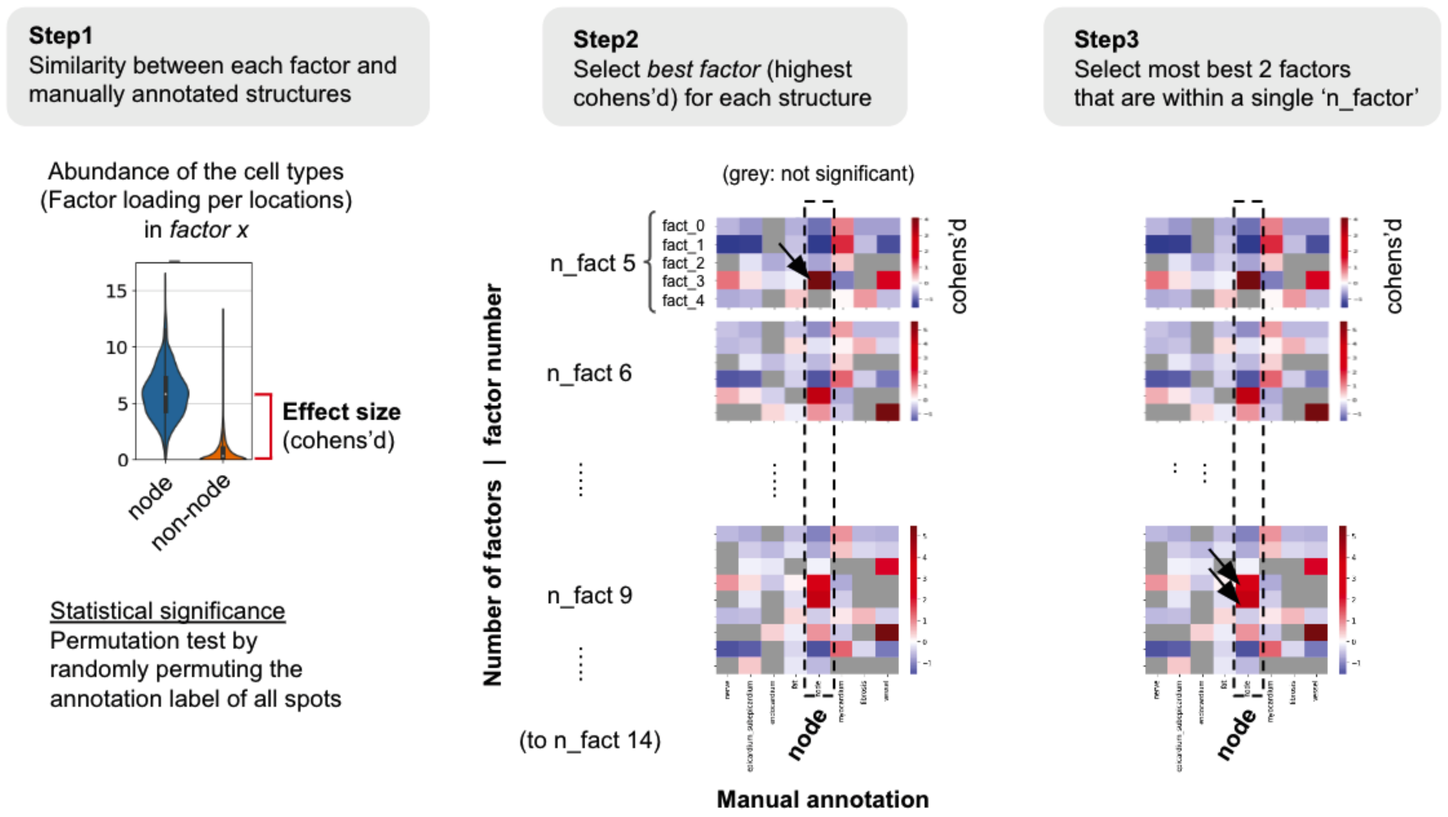

Supplementary Fig. 4 Decomposing manual structural annotations to identify cellular niches.
The steps of the similarity assessment of each factor with each manually annotated structure. Effect size (Cohen's d) of the factor loadings of the spots in a given structure against spots in the other areas is calculated (step 1). For each given structure, the factor which has the highest significant effect size is selected (best-factor) (step2). Next, multiple numbers of factors within a 'n_fact' which had effect size more than an arbitrary proportion (0.5) of the best-factor (fine-factors) are selected (step3).

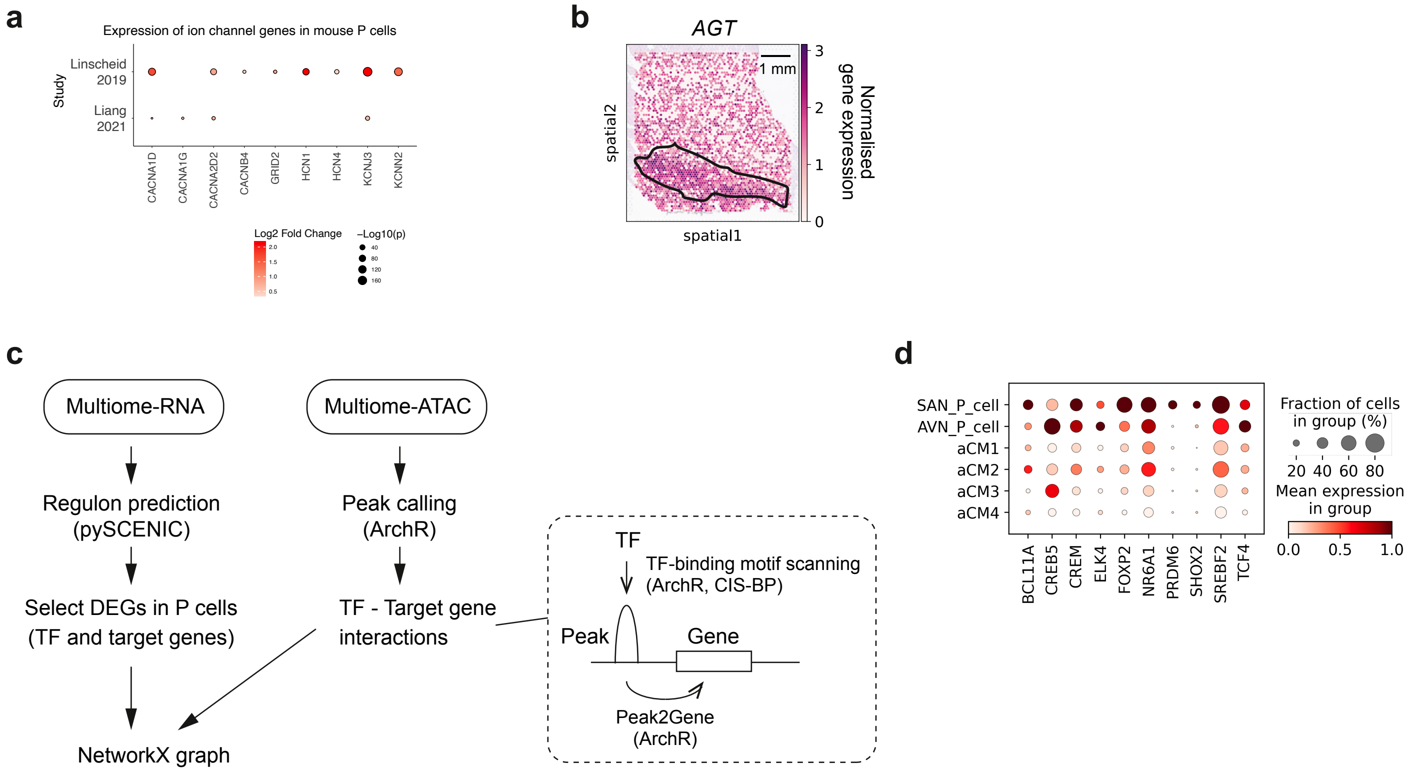

Supplementary Fig. 5 Characterisation of human pacemaker cells and their niche.

a. Mouse P cell profile. Differential expression statistics (log_2FC and -log(p-value), two-sided Wilcoxon rank-sum test) for ion channel genes which were upregulated in human SAN_P_cells relative to working atrial myocytes. p-values were adjusted for multiple comparisons using the Benjamini-Hochberg method. Plotted statistics were taken from two mouse studies (Liang 2021, Linscheid 2019) (i.e. no direct human-mouse expression comparison). No data point means no statistics were available in mouse data for that gene.

b. Visium plot showing *AGT* expression across a SAN region slide.

c. Workflow for selecting regulator TF and target gene interactions (Fig. 3c, Extended Data Fig. 8i). The network was constructed based on gene expression data and pySCENIC analysis. The interactions obtained from ATAC data were used for highlighting the interactions (Methods).

d. Dot plot showing gene expressions of regulator TF in the activator network (Fig. 3c) in multiome data.

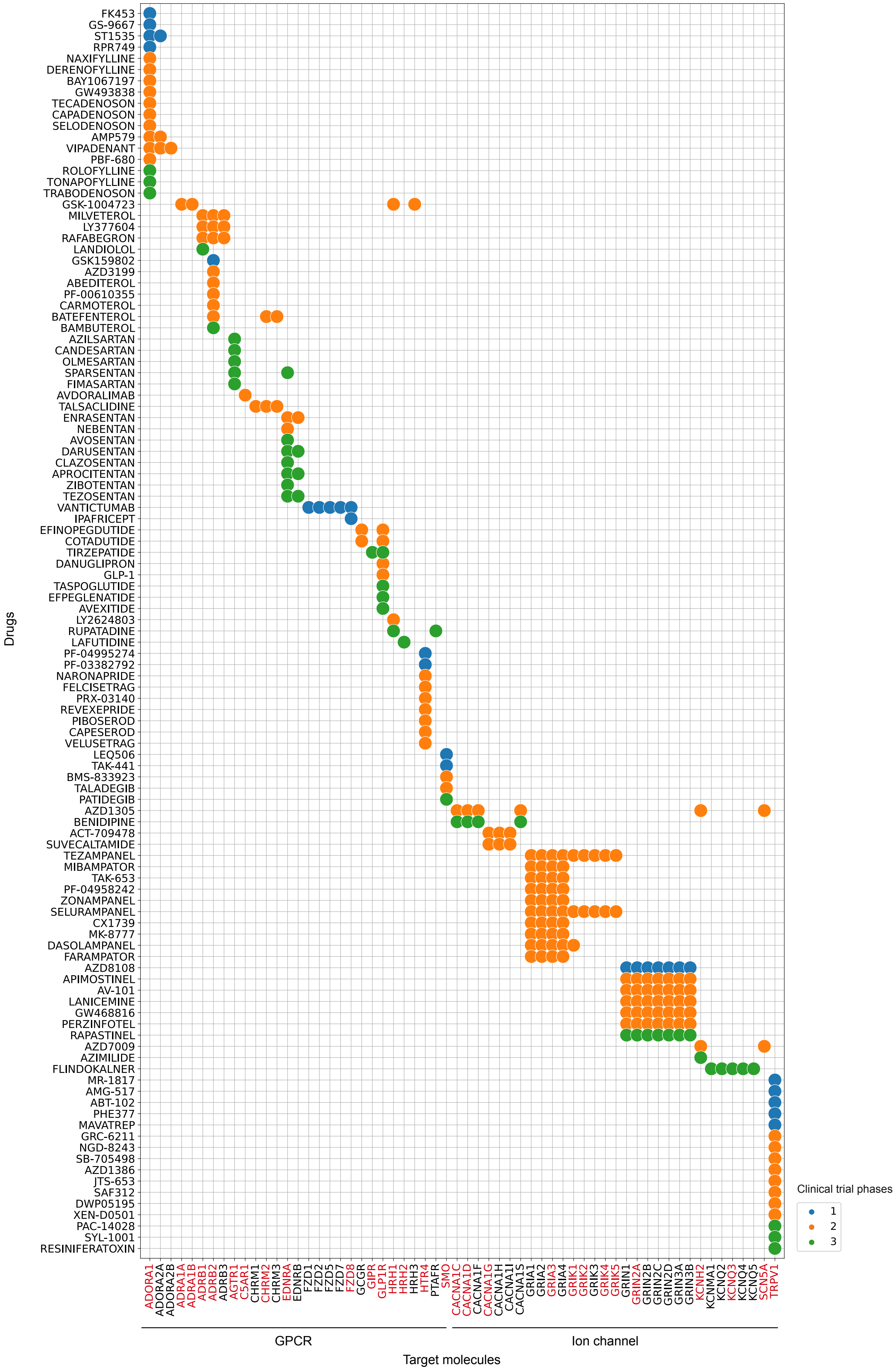

Supplementary Fig. 6 Drug target exploration at the single-cell level.
Dotplot shows drugs (y-axis) which target GPCRs or Ion channels and had higher scores in P cells compared to other cell states. Dot colours show the maximum phase of the clinical trials for each drug. Genes encoding their molecular targets are shown on the x-axis. Target genes expressed in ≥10% of P cells are highlighted in red.

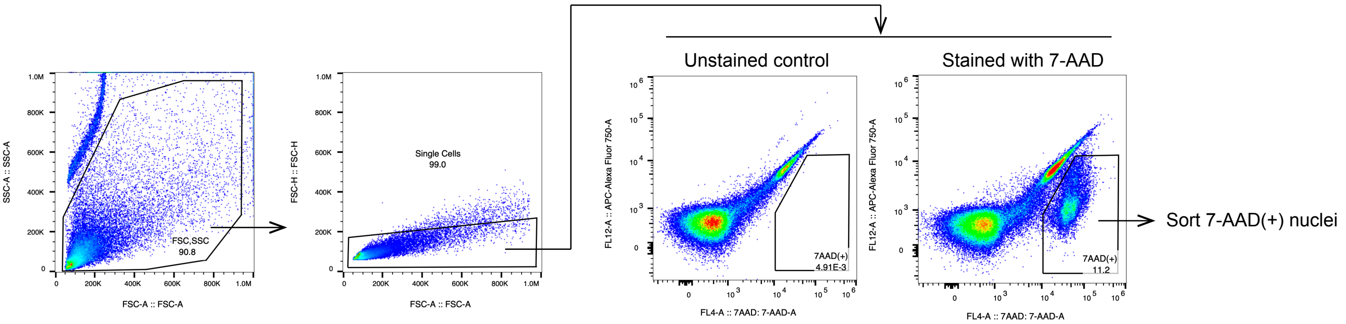

Supplementary Fig. 7 Gating strategy of flow cytometry sorting of nuclei.
Nuclei were stained with 7-AAD Viability Staining Solution (BioLegend) and positive single nuclei were sorted. The gating strategy was based on forward scatter (FSC) and side scatter properties (first panel), FSC-area and height (for selecting singlets, second panel), and the fluorescence intensity of 7-AAD (third and fourth panels).
