# Supplementary material for: Spatially resolved multiomics of human cardiac niches
Source: Nature. 2023 Jul 12;619(7971):801–10. doi: 10.1038/s41586-023-06311-1 (PMC10371870; doi:10.1038/s41586-023-06311-1)
Supplement: Supplementary file 2 — Reporting Summary [file 41586_2023_6311_MOESM2_ESM.pdf]

nature portfolio

Corresponding author(s): Sarah Teichmann
Michela Noseda

Last updated by author(s): 26th Apr 2023

Reporting Summary

Nature Portfolio wishes to improve the reproducibility of the work that we publish. This form provides structure for consistency and transparency in reporting. For further information on Nature Portfolio policies, see our Editorial Policies and the Editorial Policy Checklist.

Statistics

For all statistical analyses, confirm that the following items are present in the figure legend, table legend, main text, or Methods section.

n/a	Confirmed	
☐	☒	The exact sample size (*n*) for each experimental group/condition, given as a discrete number and unit of measurement
☐	☒	A statement on whether measurements were taken from distinct samples or whether the same sample was measured repeatedly
☐	☒	The statistical test(s) used AND whether they are one- or two-sided<br>*Only common tests should be described solely by name; describe more complex techniques in the Methods section.*
☐	☒	A description of all covariates tested
☐	☒	A description of any assumptions or corrections, such as tests of normality and adjustment for multiple comparisons
☐	☒	A full description of the statistical parameters including central tendency (e.g. means) or other basic estimates (e.g. regression coefficient) AND variation (e.g. standard deviation) or associated estimates of uncertainty (e.g. confidence intervals)
☐	☒	For null hypothesis testing, the test statistic (e.g. *F*, *t*, *r*) with confidence intervals, effect sizes, degrees of freedom and *P* value noted<br>*Give P values as exact values whenever suitable.*
☒	☐	For Bayesian analysis, information on the choice of priors and Markov chain Monte Carlo settings
☒	☐	For hierarchical and complex designs, identification of the appropriate level for tests and full reporting of outcomes
☐	☒	Estimates of effect sizes (e.g. Cohen's *d*, Pearson's *r*), indicating how they were calculated

Our web collection on statistics for biologists contains articles on many of the points above.

Software and code

Policy information about availability of computer code

Data collection	Software used include: Single-nuclei were sorted using MA900 Multi-Application Cell Sorter (Sony) and its proprietary software (Cell Sorter Software v3.1.1). Single-cell and single-nuclei RNA-seq samples were aligned using 10x Genomics CellRanger (3.0.2) or STARsolo (2.7.3a). Multiome samples were aligned using 10x Genomics CellRanger ARC (v.2.0.0). Visium spatial transcriptomics samples were aligned using 10x Genomics SpaceRanger (v.1.1.0).
Data analysis	Single cell data analysis was performed using Python (version 3), Pandas (v.1.3.5), NumPy (v.1.21.5),<br>Matplotlib (v.3.5.2), and ScanPy (v.1.8.2 and v.1.9.1). Ambient mRNA was removed using CellBender (v.0.2.0).<br>Doublet removal using Scrublet (v.0.2.1). Batch correction- scVI (v.0.14.5). ATAC data analysis using ArchR (v1.0.2) and peakVI (v.0.19.0).<br>Spatial mapping of cells using cell2location (v.0.1). CellPhoneDB (v3.0) with neuroGPCR module as described in the Method section. Gene regulatory network analysis using pySCENIC (v.0.11.2). Drug target analysis using Drug2cell (v.0.0.1)(https://github.com/Teichlab/drug2cell).<br>Quantification of calcium imaging videos was performed using FIJI (v.2.1.0) .<br>Additional custom codes used in this manuscript is available at https://github.com/kazukane/HCA_Heart_ver2.

For manuscripts utilizing custom algorithms or software that are central to the research but not yet described in published literature, software must be made available to editors and reviewers. We strongly encourage code deposition in a community repository (e.g. GitHub). See the Nature Portfolio guidelines for submitting code & software for further information.

Data

Policy information about availability of data

All manuscripts must include a data availability statement. This statement should provide the following information, where applicable:

- Accession codes, unique identifiers, or web links for publicly available datasets
- A description of any restrictions on data availability
- For clinical datasets or third party data, please ensure that the statement adheres to our policy

Open access datasets are available from ArrayExpress (www.ebi.ac.uk/arrayexpress), with accession numbers E-MTAB-12916 (Multiome snRNA-seq), E-MTAB-12919 (Multiome snATAC-seq), and XX (Visium).
Processed data of sc/snRNAseq and Visium data are available for browsing gene expression and download via heartcellatlas.org (https://www.heartcellatlas.org/#ver2)(User: heart, Password: ver2, the link will be publically available at the time of publication). A CellTypist model trained on this atlas is available for download from https://www.heartcellatlas.org/#ver2 for automated cell type annotation of other cardiac sc/snRNA-seq datasets. CellPhoneDB NeuroGPCR expansion module is available from Supp. Table 3 and 4 or https://github.com/ventolab/CellphoneDB (CellPhoneDB-database, v4.1).
The external adult heart sc/snRNA-seq dataset is available from the Human Cell Atlas Data Coordination Platform with accession number: ERP123138. The human reference genome (GRCh38) used for read mapping is available from 10X Genomics website (https://support.10xgenomics.com/single-cell-gene-expression/software/release-notes/build).
The ChEMBL database used for drug2cell analysis is available from https://ftp.ebi.ac.uk/pub/databases/chembl/ChEMBLdb/releases/chembl_30.

Human research participants

Policy information about studies involving human research participants and Sex and Gender in Research.

Reporting on sex and gender	The cohort consisted of 11 male (D2, D3, D6, D7, D8, H2, H3, H4, AV10, AV14, AH2) and 14 female (D1, D4, D5, D11, H5, H6, H7, A61, AH1, AH5, AH6, AV1, AV3, AV13) donors.
Population characteristics	Tissues are from 25 individuals, six (H2-7) were collected in North America, the remainder being collected in the United Kingdom. Donors were 20-75 years of age. 12 donors were classified as DCD (Donation after Circulatory Death: D2,D4-8,D11,A61,AH1,AH2,AH5,AV3) and 13 donors were classified as DBD (Donation after Brain Death: D1,D3,H2-7,AH6,AV1,AV10,AV13,AV14,).
Recruitment	Tissues were obtained from healthy transplant organ donors. Hearts were retrieved by trained cardiac surgeons using clinical transplant retrieval protocols. Cardiovascular history was unremarkable for all donors. We believe this method of recruitment does not introduce any bias that can impact our results.
Ethics oversight	All heart tissues were obtained from deceased transplant organ donors after Research Ethics Committee approval and informed consent from the donor families.<br><br>Research Ethics Committee approval references: 15/EE/0152 (East of England Research Ethics Committee, for D1-8,D11,A61), 16/LO/1568 (London Research Ethics Committee, for AH1,AH2,AH5,AH6) and 16/NE/0230 (North East Research Ethics Committee, for AV1,AV3,AV10,AV13,AV14).<br><br>Failing hearts samples used for validation were obtained under the Research Ethic Committee approval given to the Royal Brompton & Harefield Hospital Cardiovascular Research Centre Tissue Bank (REC ref: 19/SC/0257).

Note that full information on the approval of the study protocol must also be provided in the manuscript.

Field-specific reporting

Please select the one below that is the best fit for your research. If you are not sure, read the appropriate sections before making your selection.

☒ Life sciences ☐ Behavioural & social sciences ☐ Ecological, evolutionary & environmental sciences

For a reference copy of the document with all sections, see nature.com/documents/nr-reporting-summary-flat.pdf

Life sciences study design

All studies must disclose on these points even when the disclosure is negative.

Sample size	Sample size were determined by availability of donors within the sampling time-frame. No statistical methods were used to calculate appropriate sample size. We followed standards in the field and Human Cell Atlas criteria.
Data exclusions	For the final count matrix, we excluded cells and spots of spatial transcriptomics samples based on pre-established criteria. Cells or nuclei for each sample were filtered for more than 200 genes and less than 20% (cells) or 5% (nuclei) mitochondrial and ribosomal reads. A Scrublet (v.0.2.3) score cut-off 0.3 of was applied to remove doublets. Visium spots of each sample were filtered for more than 500 UMI counts and 300 genes.

Replication	Multiome (paired single-nuclei RNA and ATAC sequencing) was performed on the 8 regions of heart tissue from 10 adult donors, with comparable results among the donors. Visium spatial transcriptomics was performed on the 8 regions of heart tissue from 12 adult donors, with consistencies of the results between donors.<br><br>For the micrographs in Figure 1b, 2b, 2f, 3f, 3i, 5e-f; Extended Figure 2g, 4c-d, 5b(ii), 5c(ii), 6a, 7c, 10c, and 12c are representative images, from two-six independent tissue sections (for each) with similar results.<br><br>For in-vitro iPCS-CM, experiments were performed using three independent differentiation batches with similar results.
Randomization	Randomization was not relevant due to the study design where sample collection was based on availability of transplant donors.
Blinding	For the sequencing samples, we made no comparison between discreet groups for human participants, thus blinding of investigators was not necessary.

Reporting for specific materials, systems and methods

We require information from authors about some types of materials, experimental systems and methods used in many studies. Here, indicate whether each material, system or method listed is relevant to your study. If you are not sure if a list item applies to your research, read the appropriate section before selecting a response.

Materials & experimental systems

n/a	Involved in the study	
☐	☒	Antibodies
☒	☐	Eukaryotic cell lines
☒	☐	Palaeontology and archaeology
☒	☐	Animals and other organisms
☒	☐	Clinical data
☒	☐	Dual use research of concern

Methods

n/a	Involved in the study	
☒	☐	ChIP-seq
☐	☒	Flow cytometry
☒	☐	MRI-based neuroimaging

Antibodies

Antibodies used	anti IgA1-AF488 (1:200, clone: B3506B4, SouthernBiotech, cat: 9130-30), anti IgA2-AF488 (1:200, clone: A9604D2, SouthernBiotech, cat: 9140-30), anti HCN1 (1:100, polyclonal, Alomone Labs, cat: APC-056), anti HCN4 (1:200, polyclonal, Alomone Labs, cat: APC-052), anti cTnT (1:200, clone: 13-11, Invitrogen, cat: MA5-12960), anti GLP1R (1:500, polyclonal, Alomone Labs, cat: AGR-021), anti HCN1 (1:100, clone: N70/28, Abcam, cat: ab84816), anti PLP1 (1:500, clone: EPR23504-106, Abcam, cat: ab254363),<br><br>goat anti-rabbit IgG Alexa Fluor 555 (1:1000, polyclonal, LifeTech, cat: A21428), goat anti-mouse IgG Alexa Fluor 647Plus (1:1000, polyclonal, Fisher Scientific, cat: 15627898), rabbit IgG isotype (1:200, clone: DA1E, Cell Signaling, cat: 3900S), mouse IgG1 isotype (1:200, Santa Cruz, cat: sc-3877), Goat anti-rabbit IgG Alexa Fluor 647 (1:200, polyclonal, Invitrogen, cat: A21244), Goat anti-mouse IgG Alexa Fluor 488 (1:200, polyclonal, Invitrogen, cat: A11017)
Validation	IgA1 (9130-30): validated with FLISA against human IgA1-Fc comparing with IgA2, IgG, and IgM-Fc. The data shown on the supplier website.<br>IgA2 (9130-40): validated with FLISA against human IgA2-Fc comparing with IgA1, IgG, and IgM-Fc. The data shown on the supplier website.<br>HCN1 (APC-056): validated with WB, IF, and immunocytochemistry against human, mouse, and rat HCN1. The specificity has been validated in a knockout or knockdown system. The data shown on the supplier website.<br>HCN4 (APC-052): validated with WB, IF, and immunocytochemistry against human, mouse, and rat HCN4. The specificity has been validated in a knockout or knockdown system. The data shown on the supplier website.<br>cTnT (MA5-12960): validated with WB, IF, and IHC against dog, hamster, human, mouse, pig, rat, xenopus, zebrafish cTnT. This Antibody was verified by Cell treatment to ensure that the antibody binds to the antigen. The data shown on the supplier website.<br>GLP1R (AGR-021): validated with WB, IF, and IHC against human, mouse, and rat GLP1R. The data shown on the supplier website.<br>HCN1 (ab84816): validated with WB, IHC, and flow cytometry against human, mouse, and rat HCN1. The data shown on the supplier website.<br>PLP1 (ab254363): validated with WB, IF, IHC, and immunocytochemistry against human, mouse, and rat PLP1. The data shown on the supplier website.

Flow Cytometry

Plots

Confirm that:

☒ The axis labels state the marker and fluorochrome used (e.g. CD4-FITC).

☒ The axis scales are clearly visible. Include numbers along axes only for bottom left plot of group (a 'group' is an analysis of identical markers).

☒ All plots are contour plots with outliers or pseudocolor plots.

☒ A numerical value for number of cells or percentage (with statistics) is provided.

Methodology

Sample preparation	As described in the Methods section. Briefly, the single nuclei were isolated by mechanical homogenisation and washed. The nuclei were stained with commercially available 7-AAD Viability Staining Solution (BioLegend, 420404) . The samples were kept on ice and directly loaded onto the FACS-sorter.
Instrument	MA900 Multi-Application Cell Sorter (Sony)
Software	Proprietary software of the MA900 sorter (Cell Sorter Software v3.1.1)
Cell population abundance	This is not relevant since 7AAD-positive nuclei were sorted as many as possible for downstream processing.
Gating strategy	Single nuclei were selected for single signal on the SCC and FCC to avoid aggregates. The 7AAD-positive nuclei were selected.

☒ Tick this box to confirm that a figure exemplifying the gating strategy is provided in the Supplementary Information.
